# Introduction of Large Sequence Inserts by CRISPR-Cas9 To Create Pathogenicity Mutants in the Multinucleate Filamentous Pathogen Sclerotinia sclerotiorum

**DOI:** 10.1128/mBio.00567-18

**Published:** 2018-06-26

**Authors:** Jingtao Li, Yanhua Zhang, Yucheng Zhang, Pei-Ling Yu, Hongyu Pan, Jeffrey A. Rollins

**Affiliations:** a College of Plant Sciences, Jilin University, Changchun, Jilin Province, People’s Republic of China; b Department of Plant Pathology, University of Florida, Gainesville, Florida, USA; University of Nebraska—Lincoln

**Keywords:** CRISPR, Cas9, filamentous fungi, functional genomics, gene disruption, necrotroph, plant pathogens

## Abstract

The necrotrophic fungal plant pathogen Sclerotinia sclerotiorum is responsible for substantial global crop losses annually resulting in localized food insecurity and loss of livelihood. Understanding the basis of this broad-host-range and aggressive pathogenicity is hampered by the quantitative nature of both host resistance and pathogen virulence. To improve this understanding, methods for efficient functional gene characterization that build upon the existing complete S. sclerotiorum genome sequence are needed. Here, we report on the development of a clustered regularly interspaced short palindromic repeat (CRISPR)–CRISPR-associated protein 9 (CRISPR-Cas9)-mediated strategy for creating gene disruption mutants and the application of this technique for exploring roles of known and hypothesized virulence factors. A key finding of this research is that transformation with a circular plasmid encoding Cas9, target single guide RNA (sgRNA), and a selectable marker resulted in a high frequency of targeted, insertional gene mutation. We observed that 100% of the mutants integrated large rearranged segments of the transforming plasmid at the target site facilitated by the nonhomologous end joining (NHEJ) repair pathway. This result was confirmed in multiple target sites within the same gene in three independent wild-type isolates of S. sclerotiorum and in a second independent gene. Targeting the previously characterized *Ssoah1* gene allowed us to confirm the loss-of-function nature of the CRISPR-Cas9-mediated mutants and explore new aspects of the mutant phenotype. Applying this technology to create mutations in a second previously uncharacterized gene allowed us to determine the requirement for melanin accumulation in infection structure development and function.

## IMPORTANCE

Fungi that cause plant diseases by rotting or blighting host tissue with limited specificity remain among the most difficult to control. This is largely due to the quantitative nature of host resistance and a limited understanding of fungal pathogenicity. A mechanistic understanding of pathogenicity requires the ability to manipulate candidate virulence genes to test hypotheses regarding their roles in disease development. Sclerotinia sclerotiorum is among the most notorious of these so-called broad-host-range necrotrophic plant pathogens. The work described here provides a new method for rapidly constructing gene disruption vectors to create gene mutations with high efficiency compared with existing methods. Applying this method to characterize gene functions in S. sclerotiorum, we confirm the requirement for oxalic acid production as a virulence factor in multiple isolates of the fungus and demonstrate that melanin accumulation is not required for infection. Using this approach, the pace of functional gene characterization and the understanding of pathogenicity and related disease resistance will increase.

## INTRODUCTION

Sclerotinia sclerotiorum (Lib.) de Bary is a plant-pathogenic fungus characterized by necrotrophic pathogenesis on a broad range of hosts (1, 2). The penetration of healthy tissue is achieved by modified hyphae that form compound appressoria (3), and subsequent colonization is closely associated with the accumulation of oxalic acid (OA). OA has been demonstrated experimentally to be a critical virulence factor affecting the extent of colonization and tissue-macerating symptomology (4–6).

A complete genome sequence is now available for S. sclerotiorum, providing ready access to genes which are important for pathogenicity (2). Thus, developing an efficient, convenient, and economical approach for gene manipulation in S. sclerotiorum has become critical for researchers. Currently, mutants of S. sclerotiorum have been created by homologous recombination (3, 5), knockdowns by RNA interference (RNAi) (7, 8), random insertion mutations introduced by transfer DNA (T-DNA) (6, 9), and UV induction (10). The efficiency of these techniques is relatively low. Among the currently available nuclease systems for precision genome engineering, the CRISPR-Cas system has been demonstrated to be the most efficient and user friendly (11–13). CRISPR-Cas9 can site-specifically cleave double-stranded DNA, resulting in the activation of the double-strand break (DSB) repair machinery (11, 14). A relatively precise form of DSB repair is the homology-directed repair (HDR) pathway allowing for replacement mutations if a donor template with homology to the targeted locus is supplied (15). Alternatively, a DSB may be repaired by the nonhomologous end joining (NHEJ) pathway, often resulting in small insertions/deletions (indels) and point mutations (16–19). Occasional large deletions or the insertion of several hundred nucleotides at targeted DSB sites are reported to occur during NHEJ repair (20, 21).

The application of CRISPR-Cas systems to efficiently edit genomes has changed the course and speed of gene manipulation in diverse eukaryotic organisms (12). Recently, CRISPR-Cas9 technology has been successfully applied in a diversity of fungi, including *Agaricus* bisporus (22), Neurospora crassa (23), *Aspergillus* spp. (24–26), Trichoderma reesei (27), Pyricularia oryzae (22), Ustilago maydis (28), *Myceliophthora* species (29), *Ganoderma* (30), and Penicillium chrysogenum (31). However, its development and application have not yet been reported in the multinucleate plant pathogen S. sclerotiorum.

In this study, we developed a CRISPR-Cas9 system by modifying an existing plasmid (22) to include a hygromycin B (HygB) resistance selection marker for transformation with a single construct. We demonstrated that CRISPR-Cas9-induced DSBs can be repaired by insertion of DNA sequences through NHEJ. The efficiency of targeted mutations was tested using the oxalate biosynthesis gene *Ssoah1* (5, 6) and three different wild-type (WT) isolates of S. sclerotiorum. Genome resequencing was utilized to screen for off-target effects and to characterize the fate of DNA inserted into the target site. We also confirmed the phenotype of *Ssoah1* CRISPR-Cas9 insertion mutants by oxalic acid (OA) assays and characterized new phenotypes of *Ssoah1* gene disruption by comparison with knockout mutants obtained from homologous recombination. To apply this technology, we created *Sspks13* disruption mutants using this CRISPR-Cas9 system and characterized the loss of compound appressorium pigmentation phenotype in the resulting mutants.

## RESULTS

### Construction of and transformation with pCRISPR-Cas9-TrpC-Hyg.

A plasmid vector encoding the Cas9 nuclease and carrying a target site chimeric single guide RNA (sgRNA) targeting the oxalic acid biosynthesis gene (*Ssoah1*) and a dominant selectable marker (*hph*) was constructed from existing plasmids and oligonucleotides (see Materials and Methods). Following genetic transformation, two classes of transformants based on growth vigor could be distinguished on selective medium (see Fig. S1A in the supplemental material). Vigorous, nonpigmented or lightly pigmented colonies were considered hygromycin resistant (HygR), and poorly growing, pigmented colonies were considered hygromycin tolerant. Following three rounds of hyphal tip purification, transformants were screened on potato dextrose agar (PDA) supplemented with the pH indicator dye bromophenol blue (BPB). This screen was designed to distinguish loss-of-function *Ssoah1* mutants which do not accumulate oxalic acid and fail to acidify the growth medium from nonmutant transformants. Strains unable to acidify the medium (i.e., the medium remained blue) were found only among the hygromycin-resistant class of transformants (Fig. S1B). These colonies, as well as HygB-resistant transformants that were able to acidify the growth medium, were selected for further molecular and phenotypic characterization.

10.1128/mBio.00567-18.2 FIG S1  CRISPR-Cas9 mutagenesis process applied to S. sclerotiorum. (A) Regeneration of transformants, further selection, hyphal tip purification, and mutant identification. Hygromycin B (HygB) resistant (R), tolerant (T), and sensitive (S) transformants. (B) Representative transformant screen on PDA supplemented with bromophenol blue (BPB) and HygB. Mutants fail to acidify (blue) and nonmutants acidify (yellow) the growth medium. Download FIG S1, TIF file, 0.7 MB. Copyright © 2018 Li et al. 2018 Li et al. This content is distributed under the terms of the Creative Commons Attribution 4.0 International license.

### Frequency of targeted mutation identified by phenotype.

**FIG 1:**
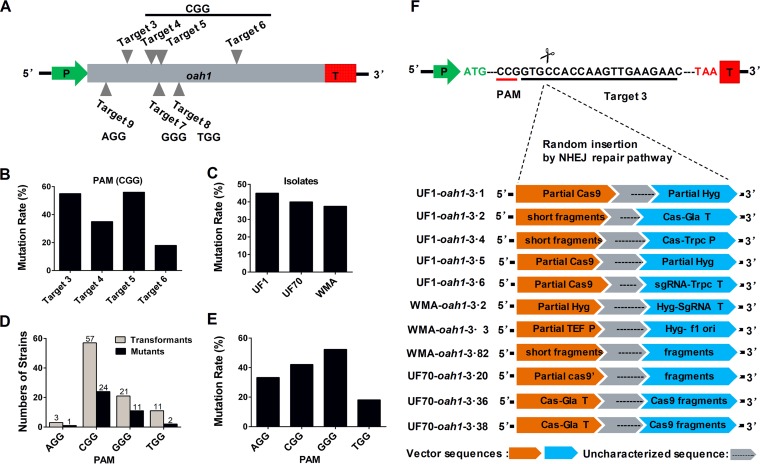
Mutation rate and characteristics of *Ssoah1* CRISPR-Cas9 target site mutations. (A) Locations of sgRNA target sites within the *Ssoah1* coding sequence. (B) Mutation rates across target sites with the same PAM sequence (“CCG”). (C) Average mutation frequency among wild-type isolates. (D) Mutant recovery by PAM sequence. (E) Frequency of mutant recovery by PAM site. (F) TAIL-PCR sequencing results demonstrating target site disruption by variable rearranged transformation vector (pCRISPR-Cas9-TrpC-Hyg) fragments.

We assessed the efficiency of the S. sclerotiorum CRISPR-Cas9 system by designing four different sgRNAs with the same CGG protospacer adjacent motif (PAM) and three additional sgRNA constructs with different PAMs (AGG, GGG, and TGG) within the coding sequence of the *Ssoah1* gene (Fig. 1A). The target sites were determined to be unique following BLASTN analysis against the S. sclerotiorum genome. In contrast to the wild type, where the color of the pH indicator growth medium changes from blue to yellow due to acidification from oxalic acid (OA) production, indicator medium colonized by a significant number of the pCRISPR**-**Cas9-Trpc-Hyg transformants with *Ssoah1* target sites remained blue (Fig. S1B). Therefore, preliminary mutagenic targeting efficiency could be easily discerned from the phenotypic ratios of blue and yellow colonies. All empty vector transformants of UF1 acidified growth medium, and two (UF1-EV2 and UF1-EV24) were chosen as controls in functional studies.

A total of 92 hygromycin-resistant transformants were obtained among the three wild-type isolates. Thirty-eight (41%) of these transformants failed to acidify the growth medium as determined from BPB-supplemented plates (Fig. S2A). Phenotypic mutants were recovered for each PAM site (Fig. 1B and S2A). The recovery of these mutants exhibited similar efficiencies (ranging from 38% to 45%) among the three S. sclerotiorum isolates tested (Fig. 1C and S2A). Transformation efficiency and mutational frequency varied across target sites with different PAM sites (Fig. 1D), but the level of variation among different PAM sequences (Fig. 1E) was similar to that of transformants with the CGG PAM sequence across different target sequences (Fig. 1B).

10.1128/mBio.00567-18.3 FIG S2  PCR verification of OA-minus mutant recovery rate. (A) Frequency of OA-minus mutants identified by bromophenol blue (BPB) plate screening. (B) Twenty-nine positive transformants obtained from three different wild-type isolates were selected for PCR verification using genomic DNA template. Primers F, R, F6, and R6 (Table S1) were used for amplification across double-stranded break target sites. Primers PycF and PycR (Table S1), specific to the *Sspyc1* gene (GenBank accession number XM_001586211) were used as a positive PCR control (*pyc*). Download FIG S2, TIF file, 0.4 MB. Copyright © 2018 Li et al. 2018 Li et al. This content is distributed under the terms of the Creative Commons Attribution 4.0 International license.

### Large insertions introduced at target sites by NHEJ.

**FIG 2:**
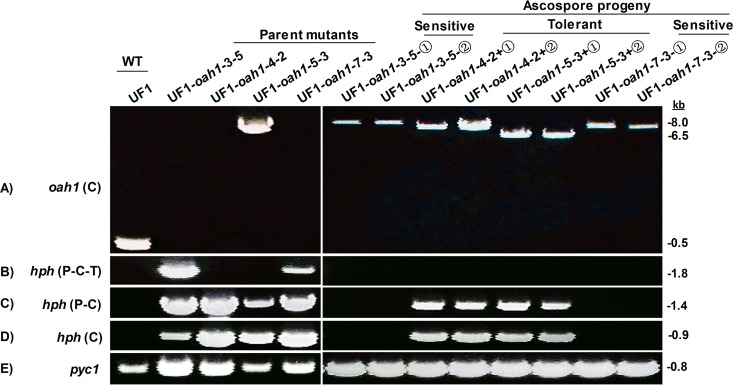
PCR verification of meiotic deletion events. (A) Long-amplification PCR of parental mutants (e.g., UF1-*oah1*-3-5 [wild-type “UF1” background, *Ssoah1* target site 3, strain number 5]) and single-ascospore progeny (+①, +②, -①, and -②) with primer pair F and R. (B to D) Long-amplification PCR with primer pair Hyg-P-F and Hyg-T-R (B), primer pair Hyg-P-F and Hyg-C-R (C), and primer pair Hyg-C-F and Hyg-C-R (D). (E) Primers PycF and PycR specific to the *Sspyc1* gene were used as a positive PCR control (*pyc1*). Primer sequences are given in Table S1.

Further molecular confirmation of *Ssoah1* mutations was initially conducted through a PCR screen. Genomic DNA from 29 of the phenotypically identified mutants was PCR amplified (Fig. S2B) using primers F and R or F6 and R6 (Table S1). All of the examined phenotypic mutants failed to amplify across the target sequence, in contrast to the wild-type and empty vector controls, which produced amplicons of the expected size. Genomic DNA sequences upstream and downstream of target 3 were also examined by PCR amplification to determine if large deletions had occurred. Amplicons upstream and downstream of the target sites were obtained for all examined mutants, and these sequences were identical to the wild type (data not shown). This result indicated that large sequence insertions at the target site were the likely cause for failed PCR amplification across target sites. Long-amplification PCR techniques were attempted in order to amplify the intervening sequences (Fig. 2A). An amplicon was obtained for only one of the 38 CRISPR mutants, UF1-*oah1*-5-3 (wild-type “UF1” background, *Ssoah1* target site 5, strain number 3). This amplicon was 6.5 kb; inserted sequences in all other mutants were presumably larger.

10.1128/mBio.00567-18.9 TABLE S1  Primers used in this research. Download TABLE S1, DOCX file, 0.02 MB. Copyright © 2018 Li et al. 2018 Li et al. This content is distributed under the terms of the Creative Commons Attribution 4.0 International license.

To further characterize sequences associated with target sites, thermal asymmetrical interlaced PCR (TAIL-PCR) and subsequent amplicon sequencing were pursued. Among 11 tested mutants with the same sgRNA (target 3) from three S. sclerotiorum isolates, all target sites contained rearranged vector segments (Fig. 1F). TAIL-PCR amplicons were limited to less than 500 bp; therefore, full sequence makeup and size of vector insertions were not determined by this approach. The sequence data revealed that some insertions were contiguous with the vector and others were comprised of rearranged segments of the transformation vector. These rearranged sequences are labeled “fragments” in Fig. 1F. We also confirmed vector insertions in two more targets (target 4 and target 5) using TAIL-PCR and DNA sequencing (Fig. S3). In all cases, gene disruption at the target site with large rearranged vector sequences without deletion of genomic DNA sequences was observed (data not shown).

10.1128/mBio.00567-18.4 FIG S3  TAIL-PCR characterization of sequences inserted at *Ssoah1* target sites 4 and 5. Sequences recovered from the 5′ and 3′ sides of the target site all matched the transformation vector (pCRISPR-Cas9-TrpC-Hyg) and are indicated by their encoded component. Download FIG S3, TIF file, 0.2 MB. Copyright © 2018 Li et al. 2018 Li et al. This content is distributed under the terms of the Creative Commons Attribution 4.0 International license.

### Effect of CRISPR-Cas9 expression on S. sclerotiorum growth and development.

Since the CRISPR-Cas9 system will be used for functional gene studies, it is imperative that the Cas9 system itself should not be deleterious to the growth or development of S. sclerotiorum. For this, we assessed growth and development of the transformants on standard PDA growth medium and PDA with 100 mg/liter HygB. The empty vector transformants (UF1-EVs) were all HygB resistant and grew and developed like the wild type (Fig. S4). All *oah1* mutants grew at the same rate as the wild type (data not shown) but formed more sclerotia toward the interior of the plate than the wild type (Fig. S4). HygB resistance, however, was variable, and the UF1-*oah1*-5-3 mutant showed only partial resistance compared to other transformants. We designated this mutant HygB tolerant, in contrast to the wild-type isolate, which was HygB sensitive, and all other mutants, which were hygromycin resistant (Fig. S4). Following multiple transfers on and off selection with no changes in phenotype, we conclude that transformants were vegetatively stable and the empty vector transformation did not have an adverse effect on S. sclerotiorum growth or development, making it useful for functional gene analysis.

10.1128/mBio.00567-18.5 FIG S4  Growth and sclerotium development phenotypes of *Ssoah1* CRISPR-Cas9 mutants and controls following 5 and 10 days after inoculation (DAI) of PDA medium and on PDA medium supplemented with 100 µg/ml HygB. Note that the wild-type isolate (UF1) is HygB sensitive. The empty vector controls (UF1-EV-2 and -24) are hygromycin B (HygB) resistant, and *Ssoah1* mutants of various target sites (target 3, UF1-*oah1*-3-5 and -6; target site 4, UF1-*oah1*-4-2 and -4; and target site 7, UF1-*oah1*-7-3) are HygB resistant. Target site 5 mutant UF1-*oah1*-5-3 is HygB tolerant. Download FIG S4, TIF file, 1.1 MB. Copyright © 2018 Li et al. 2018 Li et al. This content is distributed under the terms of the Creative Commons Attribution 4.0 International license.

### Meiotic sequence deletion and instability of HygB resistance.

Next, we tested the meiotic stability of the vector sequences inserted at the target site. Initial screens of single-ascospore progeny on selective medium indicated that HgyB resistance in ascospores from genetically pure primary transformants was unstable but the OA-minus phenotype was stable (Table 1). Almost all ascospore colonies (99%) of *Ssoah1* disruption mutants originating from multiple independent apothecia lost HygB resistance. Of these, 26% were HygB tolerant, 73% were HygB sensitive, and only one ascospore (*n* = 96) from UF1-*oah1*-7-3 retained HygB resistance. When the empty vector transformants (UF1-EVs) from two independent transformation lines were examined (*n* = 60), full HygB resistance was preserved with 55% of the ascospore progeny from UF1-EV2 and 97% of the progeny from UF1-EV24. In the HygB-tolerant parent strain UF1-*oah1*-5-3, 50% of the ascospore progeny remained tolerant and 50% were sensitive.

**TABLE 1:** Oxalic acid-minus and HygB phenotypes of meiotic progeny^a^

Strain	Parent mutant HygB phenotype	No. BPB positive/total no. tested	No. of progeny tested	No. of progeny with HygB phenotype/total no. tested		
				HygB R	HygB T	HygB S
UF1-*oah1* mutant						
3-5	R	22/22	26	0/26	0/26	26/26
3-6	R	16/16	32	0/32	2/32	30/32
4-2	R	13/13	17	0/17	17/17	0/17
4-4	R	No test	1	0/1	1/1	0/1
5-3	T	6/6	10	0/10	5/10	5/10
7-3	R	6/6	10	1/10	0/10	9/10
						
TTL		63/63	96	1/96	25/96	70/96
						
UF-EV						
EV2	R	0/6	22	12/22	0/22	10/22
EV24	R	No test	38	37/38	0/38	1/38
WT UF1	S	No test	4	0/4	0/4	4/4

a Abbreviations: R, resistant; T, tolerant; S, sensitive; TTL, total progeny tested; BPB positive, strain changed bromophenol blue medium from blue to yellow.

To determine if the shift in HygB sensitivity was associated with meiotic sequence deletions, additional PCR-based characterizations were performed on two independent single-ascospore progeny from each of four independent *Ssoah1* mutants. We tested HgyB-sensitive progeny from UF1-*oah1*-3-5 and UF1-*oah1*-7-3 and HygB-tolerant progeny from UF1-*oah1*-4-2 and UF1-*oah1*-5-3. Among these parental progenitor strains, only UF-*oah1*-5-3 produced an amplicon when primers spanning the target site were used under long-amplification PCR conditions. In contrast, all meiotic progeny produced a single amplicon spanning their specific target site (Fig. 2). The 6.5-kb amplicon produced by the UF1-*oah1*-5-3 parental strain was unchanged in its two tested HygB-tolerant meiotic progeny, and they retained the parental HygB-tolerant phenotype. The intervening sequences among the progeny from UF1-*oah1*-3-5, UF1-*oah1*-4-2, and UF1-*oah1*-7-3 ranged from 6.5 kb to 8 kb (Fig. 2A). Additional amplifications to determine the fate of sequences within the *hph* cassette revealed that UF1-*oah1*-3-5 and UF1-*oah1*-7-3 parental strains contained a full-length *hph* cassette that was absent in their progeny (Fig. 2B, C, and D). The HygB resistance in parental strain UF1-*oah1*-4-2 shifted to HygB tolerance in both tested progeny. The parental UF1*-oah1*-4-2 strain and its meiotic progeny all lacked the terminator sequences downstream of the *hph* coding sequence. Additional sequences outside the *hph* cassette were deleted in the progeny but were not further characterized.

**FIG 3:**
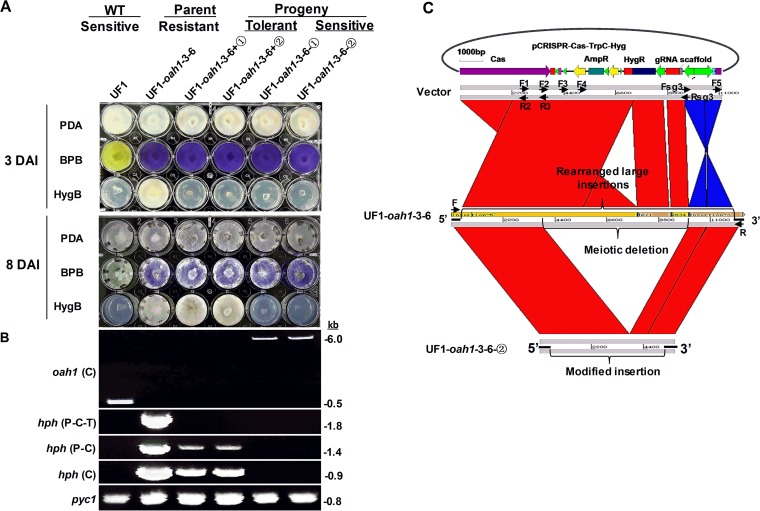
Characterization of CRISPR-Cas9-inserted sequences following meiosis. (A) Single-ascospore progeny (+①, +②, -①, and -②) of primary transformant UF1-*oah1*-3-6 (wild-type “UF1” background, *Ssoah1* target site 3, strain number 6) were assayed for OA production and hygromycin B (HygB) sensitivity on potato dextrose agar (PDA) medium supplemented with bromophenol blue (BPB) and HygB, respectively. Oxalate production (acidification) is indicated by a change in PDA-BPB medium from blue to yellow. Hygromycin B resistance (UF1-*oah1*-3-6), tolerance (UF1-*oah1*-3-6+①, +②), and sensitivity (UF1, UF1-*oah1*-3-6-①, -②) were assayed at 3 and 8 days after inoculation (DAI) of PDA-HygB medium. (B) Long-amplification PCR characterization of target site-inserted sequences in UF1, UF1-*oah1*-3-6, and single-ascospore progeny (+①, +②, -①, and -②). Primer pair F and R [*oah1* (C)] for amplification across the *Ssoah1* target site, Hyg-P-F and Hyg-T-R [*hph* (P-C-T)] for amplification of the entire *hph* cassette, primer pair Hyg-P-F and Hyg-C-R [*hph* (P-C)] for amplification of the TrpC promoter and *hph* coding sequences, and Hyg-C-F and Hyg-C-R [*hph* (C)] for amplification of the *hph* coding sequence. Primers PycF and PycR specific to *Sspyc1* were used as a positive PCR control (*pyc1*). Primer sequences are given in Table S1. (C) Global sequence alignment between the transformation vector, pCRISPR-Cas9-TrpC-Hyg, and the insertion sequence obtained from the genome sequence of primary transformant UF1-*oah1*-3-6 and its single-ascospore-derived HygB-sensitive progeny UF1-*oah1*-3-6-②. Note that three gaps exist among the assembled genomic sequence contigs of UF1-*oah1*-3-6 that presumably represent the locations of repeated unassembled sequences. Primer sequences are given in Table S1.

The results indicate that sequence deletions occur with high frequency and result in a shift from HygB resistance to HygB tolerance or sensitivity in meiotic progeny. To determine the extent of the sequence deletions, we screened two types of ascospore progeny from the UF1-*oah1*-3-6 HygB-resistant parent: HygB-tolerant (UF1-*oah1*-3-6+①, +②) and HygB-sensitive (UF1-*oah1*-3-6-①, -②). Growth on pH indicator plates confirmed that all progeny retained the OA-minus phenotype (Fig. 3A and data not shown). Further long-amplification PCR analysis revealed a 6-kb insert in the HygB-sensitive progeny and presumably longer inserts in HygB-tolerant progeny which failed to amplify (Fig. 3B). Further PCR characterization (Fig. 3B) revealed that the parental mutant UF1-3-6 retained the full *hph* cassette (promoter-*hph*-terminator), but all four ascospore progeny acquired deletions in the *hph* cassette, resulting in negative PCR results. The HygB-tolerant progeny (UF1-*oah1*-3-6+①, +②) retained the *hph* coding sequence and the TrpC promoter but suffered a deletion in the terminator sequences. HygB-sensitive progeny (UF1-*oah1*-3-6-①, -②), in contrast, had more extensive deletions and lacked the entire *hph* coding sequence.

To characterize the CRISPR-Cas9-mediated insertion event and subsequent meiotically derived deletion events in greater detail, the genome of the UF1-*oah1*-3-6 primary transformant was sequenced, assembled, and compared to the target site insertion sequence of meiotic progeny UF1-*oah1*-3-6-②. This Illumina-based genome was assembled into 5,026 contigs (≥500 bp) with an average read depth of 91. Using BLASTN to query the genomic contigs for CRISPR-Cas9-TrpC-Hyg vector sequences identified 13 independent contigs. Of these, 11 were small contigs containing only vector sequences. The remaining two large contigs contained genomic DNA-vector sequence fusions. The presence of only two contigs with vector DNA flanked by genomic DNA sequence demonstrated that insertion into the CRISPR-Cas9 target site represented the only integration site within the genome and that no off-target insertions were present within the genome. The 13 contigs were joined using DNAMAN software to produce four contiguous fragments representing the linear order of genomic and vector insert sequences. Three sequence gaps in the vector insert appear to be the result of duplicated segments that could not be properly assembled. This is supported by the approximately 34-fold-higher sequence depth of vector sequence contigs relative to flanking genomic sequence contigs and our inability to PCR amplify across the vector insertion site.

Alignment of the sequences inserted into the UF1-*oah1*-3-6 genome with the sequence of the transformation vector demonstrated that the vector sequences inserted into the genome were rearranged relative to the original transformation vector (Fig. 3C). Alignment of the insert sequence assembly of UF1-*oah1*-3-6 to the amplicon sequence of progeny UF1-*oah1*-3-6-② indicated that the meiotic insert sequence deletion occurred within the interior of the large insertion (Fig. 3C). This deletion included the *hph* cassette but retained 5.7 kb of vector sequence within the insertion site. These results were validated by additional PCR analysis shown in Fig. S5.

10.1128/mBio.00567-18.6 FIG S5  PCR verification of the UF1-*oah1*-3-6 and UF1-3-6-② CRISPR-Cas9-inserted sequences. To test the validity of the insertion sequence structure depicted in Fig. 3B, PCR amplifications were performed within the insertion and across the insert-genomic DNA junction in both the primary transformant, UF1-*oah1*-3-6, and its meiotic progeny, UF1-*oah1*-3-6-②. Amplification using flanking genomic DNA primer “F” and vector primers “R2” and “R3” (Fig. 3B) validated the 5′ insertion junction of UF1-*oah1*-3-6 obtained from TAIL-PCR (Fig. 1) and the genome assembly (A). Likewise, amplification with flanking genomic DNA primer “R” and vector sequence Rsg3 validated the 3′ insertion junction from UF1-*oah1*-3-6 (A). Additional PCR results using vector-vector and vector-genomic DNA primers indicated that the actual insertion was larger than the 11-kb vector sequence (B and C). Using vector primers F1, F2, F3, and F4 with the 3′ genomic DNA R primer produced only an amplicon with the F4×R combination; however, when the F4 primer was used alone, an amplicon of the same size, ~9.5 kb, was produced. These results indicate the presence of large (>10-kb) intervening sequences, including a second copy of the F4 primer on the opposite strand of the inserted DNA (B). PCR amplification results with the F1, F2, F3, and F4 primers paired with Fsg3 as the reverse primer produced expected ~4- and ~5-kb amplicons for F4×Fsg3 and F2×Fsg3, respectively, but a much smaller, ~1.5-kb, amplicon for F1×Fsg3 indicative of the presence of a second copy of the sequence encoding the F1 primer (C). Thus, the vector DNA insertion in UF1-*oah1*-3-6 was much larger and more complex than we could ascertain from Illumina genome sequence assembly and PCR. This is likely due to the insertion of multiple duplicated sequences. The insert sequence amplicon from the UF1-*oah1*-3-6-② meiotic progeny was fully sequenced by Sanger sequencing primer walking and verified by PCR amplification (C). The vector sequences adjacent to the 5′ and the 3′ genomic DNA target site were preserved, but most of the intervening vector sequences were absent, likely due to deletion mediated by homologous recombination between sequence repeats during meiosis. Primer sequences are given in Table S1. Download FIG S5, TIF file, 0.2 MB. Copyright © 2018 Li et al. 2018 Li et al. This content is distributed under the terms of the Creative Commons Attribution 4.0 International license.

### Disruption mutation of *Ssoah1* blocks OA accumulation and affects sclerotium development.

**FIG 4:**
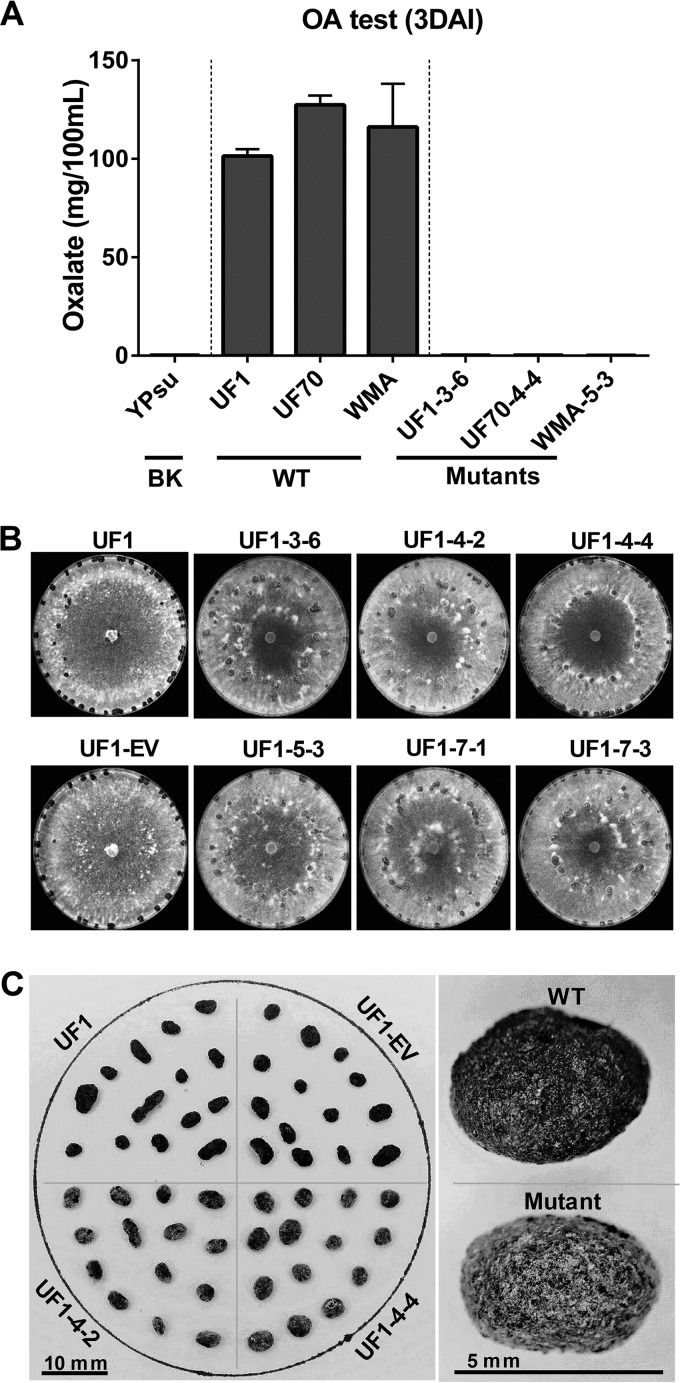
Quantification of oxalic acid and assessment of sclerotium development in CRISPR-Cas9 mutants. (A) Quantification of oxalic acid 3 days after inoculation (DAI) and growth on yeast-phosphate-sucrose (YPSu) medium. Three CRISPR-Cas9-derived *Ssoah1* mutants (UF1-3-6, UF70-4-4, and WMA-5-3) were compared to their wild-type progenitors (UF1, UF70, and WMA, respectively) and an uninoculated medium blank (BK). (B) Growth and pattern of sclerotium development of six independent CRISPR-Cas9-derived *Ssoah1* mutants (UF1-3-6, -4-2, -4-4, -5-3, -7-1, and -7-3) relative to the wild-type progenitor (UF1) and empty vector control (UF1-EV) on PDA 10 DAI. (C) Size, shape, and color characteristics of sclerotia from two independent CRISPR-Cas9-derived *Ssoah1* mutants (UF1-4-2 and -4-4) and their wild-type (UF1) and empty vector (UF1-EV) controls.

Having established the basis of the mutagenic events occurring in our CRISPR-Cas9 strains, we turned our attention to characterizing the resulting phenotypes in greater detail. When cultured with pH-indicating dye, all *oah1* mutant strains failed to acidify the growth medium, remaining 1.0 to 1.5 pH units above wild-type cultures (Fig. 3A and S1 and data not shown). As CRISPR-Cas9 created insertion mutants rather than gene deletion mutations, the accumulation of oxalic acid was quantified to determine if *oah1* mutants with different insertion target sites all resulted in loss of Oah1 function. Wild-type isolates accumulated a high level of OA, approximately 1 mg/ml, and no OA accumulation was detected in any of the *Ssoah1* CRISPR-Cas9 mutants (Fig. 4A and data not shown).

The CRISPR-Cas9 mutations also affected sclerotium development and distribution. In small (1.5-cm) PDA cultures, OA-minus mutants produced melanized sclerotia at 7 days after inoculation (DAI), while WT produced melanized sclerotia by 5 DAI (Fig. S6). When cultures were observed after 12 days of growth on PDA in 9-cm petri dishes, the *Ssoah1* mutants, regardless of their CRISPR-Cas9 target site, exhibited similar sclerotium development and distribution. This development differed from the wild-type UF1 and empty vector transformants (UF1-EVs) (Fig. 4B and S4). In the wild type, sclerotia were produced at or near the edge of the petri dish, whereas distribution of sclerotia from the mutants tended to be more scattered. Mature sclerotia of the *Ssoah1* mutants contained nonpigmented hyphae covering the entire sclerotial surface (Fig. 4C). The presence of consistently similar sclerotial development among CRISPR-Cas9 mutants and the previously reported *Ssoah1* knockout mutant KO7 in the WMA isolate background (6) confirms that this phenotype is the result of *Ssoah1* loss of function (Fig. S6). Although sclerotium development was affected in *Ssoah1* mutants, carpogenic germination and apothecium production proceeded along the wild-type timeline, and viable ascospores were collected (data not shown).

10.1128/mBio.00567-18.7 FIG S6  Growth and development of wild-type and CRISPR-Cas9-derived *Ssoah1* mutants. Colonies of wild-type isolates (UF1, UF70, and WMA) and their derived mutants (UF1-3-6, UF70-4-4, and WMA-5-3) were photographed 3, 5, 7, and 10 days after inoculation of potato dextrose agar (PDA) medium. Download FIG S6, TIF file, 1.6 MB. Copyright © 2018 Li et al. 2018 Li et al. This content is distributed under the terms of the Creative Commons Attribution 4.0 International license.

### Disruption of *Ssoah1* increases compound appressorium development.

**FIG 5:**
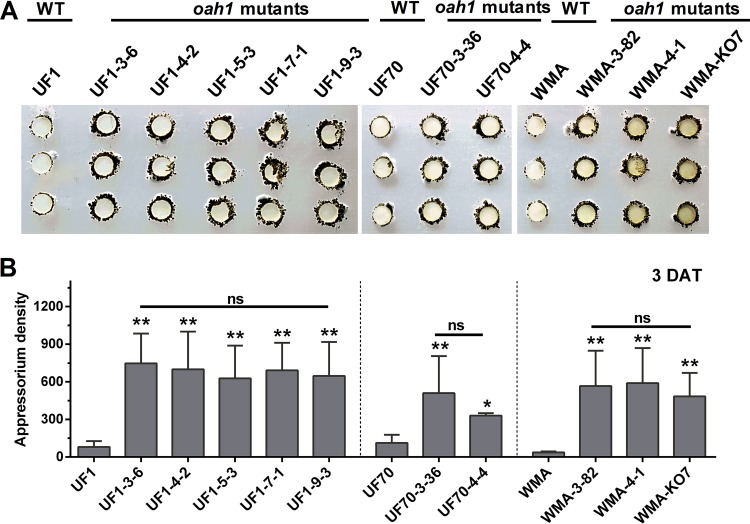
Assay and quantification of compound appressorium development in CRISPR-Cas9-derived *Ssoah1* mutants. Wild-type (WT) isolate UF1 was compared to five independent *Ssoah1* mutants (UF1-3-6, UF1-4-2, UF1-5-3, UF1-7-1, and UF1-9-3), WT isolate UF70 was compared to two independent CRISPR-Cas9-derived mutants (UF70-3-36 and UF70-4-4), and WT WMA was compared to two independent CRISPR-Cas9-derived *Ssoah1* mutants (WMA-3-82 and WMA-4-1) and the previously described *Ssoah1* gene deletion mutant WMA-KO7. Pictures were taken 3 days after transfer (DAT) of mycelium-colonized potato dextrose agar-colonized plugs to paraffin film. (B) Quantity of compound appressoria calculated by ImageJ pixel density scanning compared among the WT isolates and their derived mutants (for each strain, *n* = 6; *, *P* < 0.05; **, *P* < 0.01; ns, no significant differences at *P* = 0.05).

To assess phenotypes related to pathogenicity, compound appressorium development was initially analyzed. Three days after placement onto paraffin film, vegetative hyphae from CRISPR-Cas9 *Ssoah1* mutants formed visibly more mature pigmented compound appressoria than their wild-type progenitors (Fig. 5A). The previously characterized WMA knockout mutant KO7 (6) also displayed high-density compound appressorium development, suggesting that the excessive production of compound appressoria is related to *Ssoah1* loss of function and not the CRISPR-Cas9 system itself. The quantity of compound appressoria was calculated by density scanning and confirmed the significant increase in appressorium production by OA-minus mutants (Fig. 5B). CRISPR-Cas9-mediated *Ssoah1* disruption also caused an increase production of compound appressoria on plant surfaces, including onion epidermal strips (data not shown).

### Disruption of *Ssoah1* alters disease symptomology uniformly across genotypes.

**FIG 6:**
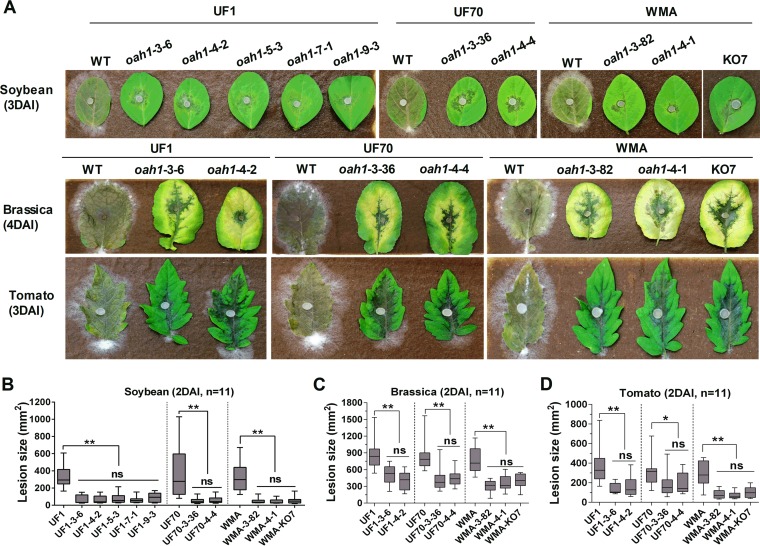
Assessment of pathogenicity and quantification of virulence in *Ssoah1* CRISPR-Cas9-derived *Ssoah1* mutants on soybean, Brassica carinata, and tomato leaves. (A) Wild-type (WT) isolate UF1 was compared to five independent *Ssoah1* CRISPR-Cas9-derived mutants (UF1-3-6, UF1-4-2, UF1-5-3, UF1-7-1, and UF1-9-3), WT isolate UF70 was compared to two independent CRISPR-Cas9-derived *Ssoah1* mutants (UF70-3-36 and UF70-4-4), and WT WMA was compared to two independent CRISPR-Cas9-derived *Ssoah1* mutants (WMA-3-82 and WMA-4-1) and the previously described *Ssoah1* gene deletion mutant WMA-KO7. Symptom development on soybean and tomato was photographed 3 days after inoculation (DAI) and 4 DAI for B. carinata (Brassica). (B to D) Quantification of lesion area on soybean (B), *Brassica* (C), and tomato leaves (D) 2 DAI with the same mutants and wild type as in panel A. Eleven inoculations were performed and quantified by image density scanning with ImageJ software for each strain (ns, not significantly different at *P* = 0.05; *, *P* < 0.01; **, *P* < 0.001).

**FIG 7:**
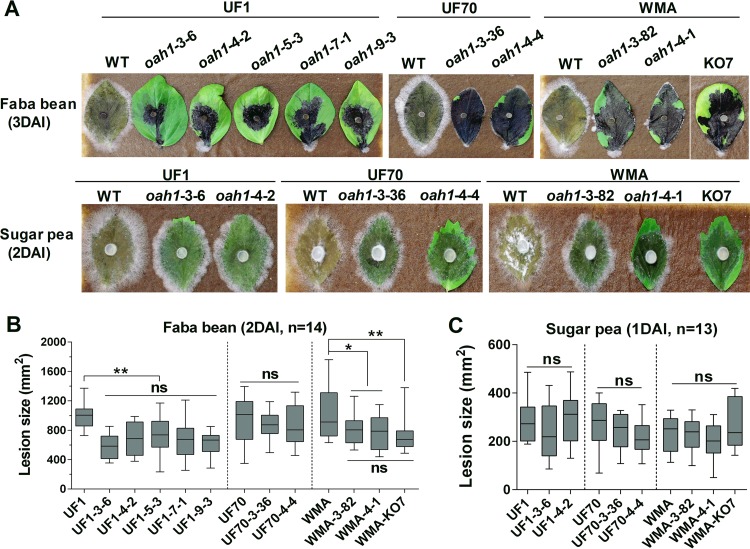
Assessment of pathogenicity and quantification of virulence in *Ssoah1* CRISPR-Cas9-derived *Ssoah1* mutants on faba bean and sugar pea. Wild-type and mutant strains were the same as in Fig. 6. (A) Symptom development on faba bean and sugar pea was photographed by 3 days after inoculation (DAI) and 2 DAI, respectively. (B and C) Lesion areas on faba bean (B) (2 DAI) (*n* = 14) and on sugar pea (C) (1 DAI) (*n* = 13) were quantified by image density scanning with ImageJ software for each strain (ns, not significantly different at *P* = 0.05; *, *P* < 0.05; **, *P* < 0.01).

Virulence was evaluated on a variety of hosts to determine if differences existed among different *Ssoah1* target site mutants and among mutants from different wild-type backgrounds. The wild-type isolates caused severe symptoms on all tested hosts (Fig. 6 and 7). The virulence of *Ssoah1* mutants was severely attenuated relative to wild type as previously reported (5, 6) but did not vary among mutation target sites or genetic background (Fig. 6 and 7). Consistent with previously published data (6), host colonization by *Ssoah1* mutants did vary by host. On detached soybean, Brassica carinata, and tomato leaves, all OA-minus mutants produced limited lesions surrounded by green or, in the case of B. carinata, yellow tissue (Fig. 6). On detached faba bean leaves, all *Ssoah1* mutants produced dark, spreading lesions that expanded slightly slower than wild-type lesions in UF1 and WMA backgrounds (Fig. 7A and B). On pea leaves, all *Ssoah1* mutants and wild-type isolates produced similar lesion sizes, but host tissue colonized by the mutants remained green relative to the brown, macerated lesions produced by the wild-type isolates (Fig. 7A and C).

### Disruption of *Sspks13* eliminates pigmentation of compound appressorium.

**FIG 8:**
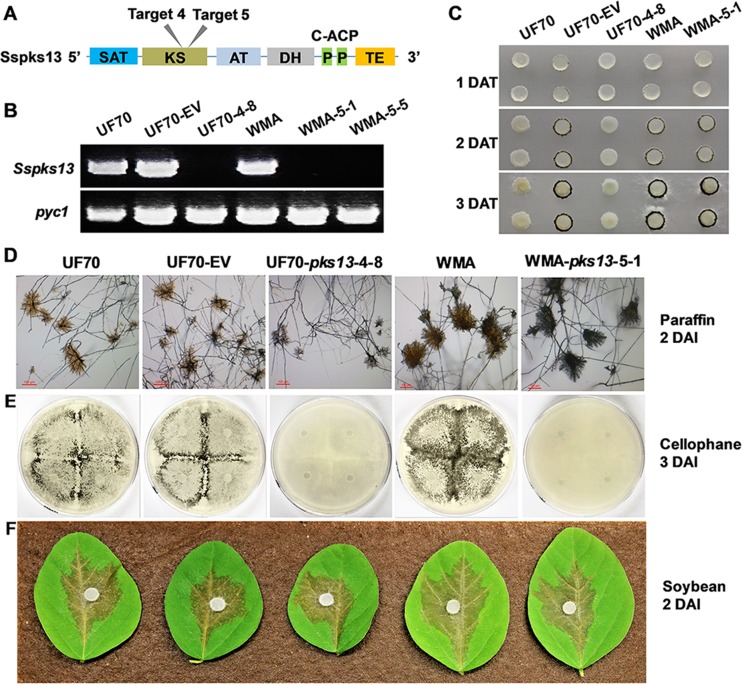
Creation and phenotypic characterization of CRISPR-Cas9-derived *Sspks13* mutants. (A) Cas9 target site locations within the encoded polyketide synthase domains of *Sspks13*. SAT, starter unit acyl carrier protein transacylase; ACP, acyl carrier protein; KS, beta-ketoacyl synthase domain; AT, malonyl coenzyme A ACP transacylase; DH, dehydratase domain; P, phosphopantetheine attachment site, an essential prosthetic group of ACP; TE, thioesterase domain. (B) Verification of *Sspks13* gene disruption by PCR of genomic DNA with primers pks13-F and pks13-R. Primers PycF and PycR specific to the *Sspyc1* gene (GenBank accession no. XM_001586211) were used as a positive PCR control (*pyc*). Primer sequences are given in Table S1. (C) Compound appressorium development by the wild-type (WT) isolate UF70 and the UF70 empty vector control strain (UF70-EV) was compared to the *Sspks13* CRISPR-Cas9-derived mutant (UF70-4-8), and that of WT isolate WMA was compared to the CRISPR-Cas9-derived *Sspks13* mutant (WMA-5-1). Pictures were taken 1, 2, and 3 days after transfer (DAT) of mycelium-colonized potato dextrose agar-colonized plugs to paraffin film. (D) Microscopic examination of compound appressoria formed on paraffin film 2 DAT. Bar, 100 µm. (E) Macroscopic assay for compound appressorium development on PDA overlaid with cellophane, 3 days after inoculation (DAI). (F) Pathogenicity and symptom development on detached soybean leaves 2 DAI.

Having demonstrated the utility of CRISPR-Cas9 for functional gene analysis with the previously characterized *Ssoah1* gene, we chose a second, previously uncharacterized, S. sclerotiorum gene for functional analysis. This gene, *Sspks13*, is predicted to encode a polyketide synthase for melanin biosynthesis. Two sgRNA target sites with “CGG” PAM sequences located within the beta-ketoacyl synthase (KS)-encoding domain of *Sspks13* were designed (Fig. 8A). Three mutants, UF70-pks13-4-8, WMA-pks13-5-1, and WMA-pks13-5-5, were obtained after HygB selection and PCR screening. All three had large DNA insertions at the target site yielding negative PCR results when attempting to PCR amplify across the target site (Fig. 8B).

Phenotypically, *Sspks13* disruption did not affect vegetative hypha growth, sclerotium formation, or apothecium production (data not shown). However, the *Sspks13* disruption appeared to inhibit the normal production of compound appressoria when assayed on paraffin film (Fig. 8C). To investigate whether *Sspks13* disruption affected compound appressorium development or only pigmentation, compound appressoria produced on paraffin film were observed microscopically (Fig. 8D). These observations revealed that *Sspks13* mutants could form structurally normal compound appressoria but that they lacked pigmentation within the amorphous matrix surrounding the appressorium. Lack of pigmentation in compound appressoria was also evident macroscopically when mutants were cultured on cellophane (Fig. 8D). We further assessed the virulence of the *Sspks13* mutants on a variety of hosts, including soybean, B. carinata, tomato, faba bean, pea, and *Arabidopsis*. On detached leaves of all hosts, the *Sspks13* disruption mutants produced lesions similar in rate of expansion size and appearance to the wild type (only results for soybean leaves 2 DAI are shown [Fig. 8D]). These results indicate that *Sspks13* CRISPR-Cas9 mutants differentiated albino compound appressoria but that virulence remained unaffected.

## DISCUSSION

We have developed a simple and efficient CRISPR-Cas9 system for targeted disruption of S. sclerotiorum genes using a single circular plasmid that provides (i) the Cas9-sgRNA targeting nuclease, (ii) a dominant selectable marker, and (iii) donor DNA for insertional mutagenesis. The insertion of nonhomologous DNA in the Cas9-mediated DSB appears to be dependent on the NHEJ pathway, as has been reported in Aspergillus fumigatus (25) and in DSB insertional techniques developed in zebra fish and human cell lines (32, 33). Previously reported mutation rates using the CRISPR-Cas9 system in Pyricularia oryzae were 36 to 84% by homologous recombination (22) and in A. fumigatus were 25 to 53% by NHEJ (25). In A. oryzae, 10 to 20% mutant recovery by NHEJ was reported (24). Low efficiency was also reported in Aspergillus carbonarius and Aspergillus luchuensis, complicated perhaps by multiple rounds of conidial selection required to isolate pure homokaryotic mutants (26). Our work demonstrated that the efficiency of targeted mutation following three rounds of hyphal tip selection in the multinucleate S. sclerotiorum ranged from 38% to 45% by NHEJ across all target and PAM sequences in three distinct isolates. Rarely, some sgRNA targets may not work, as was the case for *Ssoah1* target 2 (data not shown). Not all sites are amenable to targeting (26), and locus and cell type are known to influence DNA integration efficiency via NHEJ (34).

Repair of DSB by NHEJ is reported to create small indels in various organisms, including fungi (24–27), plants (35, 36), and mammalian cells (13, 18, 21). The creation of indels was the initial expectation for S. sclerotiorum CRISPR-CAS9-mediated transformation as well. The recovery of large vector sequence inserts in 100% of the recovered transformants suggests the existence of nuclease activity within the S. sclerotiorum transformation procedure capable of cleaving the circular transformation vector at multiple sites. Assays (see Fig. S7 and Text S1 in the supplemental material) to pinpoint the origin of this nuclease activity determined that while the lysing enzyme solution used for preparing protoplasts contained significant nuclease activity, intact protoplasts and the genetic transformation procedure itself were not significant sources of nuclease activity. The lysate obtained from protoplasts contained significant nuclease activity that varied among batches of protoplast preparations, indicating the potential for cleavage of the transforming plasmid DNA by endogenous nucleases. The nature of this nuclease activity and its endogenous function in S. sclerotiorum require further investigation. TAIL-PCR analysis of the target site sequences flanking the vector insertions did not identify any small or large sequence insertions or deletions other than the inserted vector sequences. This finding suggests precise CRISPR-Cas9 double-strand cleavage and exogenous DNA insertion without further modification of the cleaved ends. The vector-genomic DNA junctions did not reveal microhomologies that would be expected for homology-dependent repair. Based on our draft genome sequencing results of mutant UF1-*Ssoah1*-3-6, off-target sequence insertions also were not observed. In addition, all the *Ssoah1* CRISPR mutants displayed consistent phenotypes, similar to the functionally confirmed gene knockout mutant, which indicated that no traits other than oxalic acid production were altered. Utilizing the complete S. sclerotiorum genome sequence (2) as a resource for target site design may have been an important factor in decreasing the potential for off-target events.

10.1128/mBio.00567-18.1 TEXT S1 Supplemental materials and methods. Download TEXT S1, PDF file, 0.1 MB. Copyright © 2018 Li et al. 2018 Li et al. This content is distributed under the terms of the Creative Commons Attribution 4.0 International license.

10.1128/mBio.00567-18.8 FIG S7  Agarose gel electrophoresis assays for nuclease activity in components and steps of the genetic transformation procedure. (A) Plasmid DNA incubated 24 h with protoplast lysing enzyme solution. MW, DNA molecular weight marker; a, b, c, and d, four replicates of the lysing enzyme treatment; water 24 h RT is plasmid DNA incubated for 24 h at room temperature (RT); 1, replicate samples of the transformation procedure using isolate UF-1, 24 h after addition of the regeneration medium; 2, same as 1 but utilizing isolate “1980”; water + RM 24 h RT is plasmid DNA that has gone through the entire protoplast transformation procedure followed by the addition of regeneration medium and incubation for 24 h at room temperature (RT). Note that the retardation of DNA migration is due to the presence of high concentrations of polyethylene glycol and sucrose from the transformation procedure samples. (B) Treatment of plasmid DNA with lysed and unlysed protoplasts for 24 h at room temperature (RT). 1, replicate samples using isolate UF-1; 2, replicate samples using isolate “1980”; EcoRI, plasmid DNA digested with restriction enzyme EcoRI; Stock, plasmid DNA obtained directly from −20°C storage. (C) Lysed protoplast assay as in panel B but utilizing a different preparation of protoplast. Download FIG S7, TIF file, 1 MB. Copyright © 2018 Li et al. 2018 Li et al. This content is distributed under the terms of the Creative Commons Attribution 4.0 International license.

**FIG 9:**
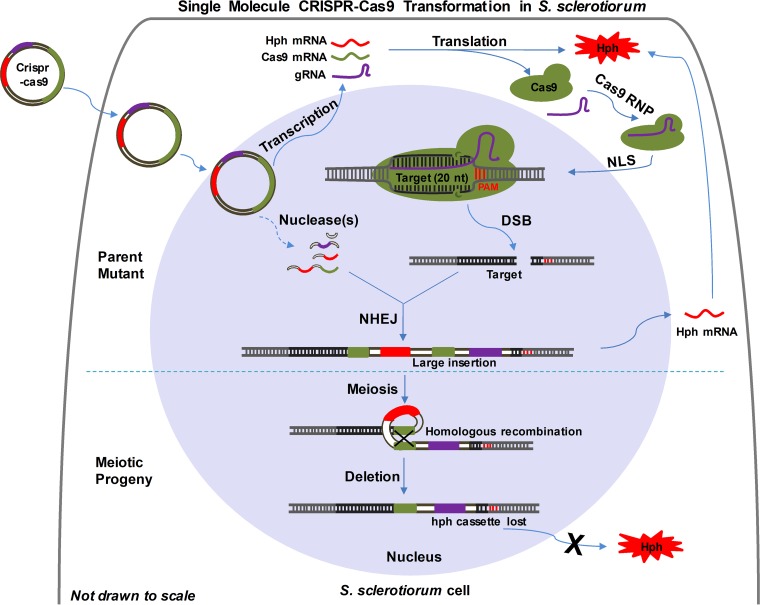
Working model of single-molecule CRISPR-Cas9 transformation in S. sclerotiorum. The top half of the diagram illustrates the transformation and vector sequence integration events as described in the text. The bottom half of the diagram illustrates the deletion of tandem, direct repeat vector sequences by intrachromosomal recombination during meiosis following self-mating. NHEJ, nonhomologous end joining; DSB, double-stranded break; NLS, nuclear localization sequence; PAM, protospacer adjacent motif; Hph, hygromycin B phosphotransferase; gRNA, guide RNA; Cas9, CRISPR-associated protein 9; RNP, ribonucleoprotein.

Our working model of the CRISPR-Cas9 system functioning in S. sclerotiorum under our experimental conditions is illustrated in Fig. 9. Following cellular uptake of the transformation vector (pCRISPR-Cas9-TrpC-Hyg), there were two different editing stages: (i) mutagenesis by NHEJ in primary transformants and (ii) sequence loss during meiosis. For the initial mutation, the vector enters the nucleus and expresses its functional elements: Cas9, sgRNA, and the selective marker *hph*. Simultaneously, or soon after, the vector is cleaved at multiple sites by an endogenous nuclease(s) and the resulting vector fragments are integrated into the targeted DSB created by the Cas9-sgRNA nuclease. This targeted integration event may incorporate multiple copies of rearranged vector sequences and is vegetatively stable. The lack of small sequence insertions or deletions typically associated with error-prone NHEJ may be the result of multiple plasmid sequences taken up by a single protoplast serving as an abundant substrate for cleavage and the repair of broken, double-stranded DNA. When individual transformants of this homothallic species are self-mated, intrachromosomal recombination further rearranges sequences separated by indirect repeats and eliminates intervening sequences between tandem direct repeats. This form of intrachromosomal recombination appears to occur at a high frequency based on the large number of HygB-sensitive progeny recovered from multiple transformants. This offers a method for recycling the selectable marker for further rounds of mutagenesis with the same vector backbone. Improvements to the efficiency of this system may be possible by replacing the *trpC* promoter with a polymerase III (PolIII) promoter to retain the sgRNA within the nucleus. Additionally, providing the selection marker as a linear DNA molecule separate from the transformation vector may provide a substrate for insertional NHEJ repair and decrease potential for ectopic marker integration at nontarget sites, thus increasing mutant recovery efficiency. These possibilities are currently under investigation.

The acquisition of large sequence inserts within double-stranded breaks is not unprecedented. Recently, a large insertion of 800 nucleotides comprised of repetitive sequences was observed in a CRISPR-edited mouse genome (21). In A. fumigatus, NHEJ-mediated events at the cut site varied from single-nucleotide indels to large insertions of linear vector DNA and PCR amplicons in as many as 50% of transformants in single-molecule transformations (25). This capacity to insert foreign DNA into DSBs has been the basis of mutational and precise engineering strategies in zebra fish and mammalian cells (32, 33). What appears unique to S. sclerotiorum is the ability to endogenously process introduced DNA (i.e., to linearize circular DNA) and effectively insert it into DSBs. This finding may serve as the basis of further improving CRISPR-Cas9-mediated manipulation of the S. sclerotiorum and other fungal genomes for mutagenic and other gene editing characterizations.

A second useful tool, selectable marker recycling, developed from the observation of HygB resistance instability in meiotic progeny. Several lines of evidence from our results suggest that multiple rearranged fragments of the vector were present in the mutants. These include the inability to PCR amplify across insertion junctions, failure to assemble vector reads into a single contig from the draft genome reads, and the observed rearrangement of vector sequences from TAIL-PCR. Assembled genome reads did not identify the full extent of the insertion site repeats, but a series of PCR amplifications utilizing vector and flanking genomic DNA sequences as primers confirmed the existence of multiple copies of rearranged vector sequences at the target sites. Premeiotic intrachromosomal recombination between direct tandem repeats eliminates intervening sequences and one copy of the duplicated sequence at a high frequency in both homothallic and heterothallic filamentous ascomycetes (37). In *Sclerotinia* species, intrachromosomal recombination between direct and indirect repeats has been shown to result in meiotic deletion and inversion, respectively, of intervening sequences at the *MAT* locus (38, 39). The frequent recovery of hygromycin-sensitive ascospore progeny from the self-mating of individual CRISPR-Cas9 transformants indicated that *hph* cassette sequence deletion occurred at high frequency as a result of duplicated vector sequences within the target sites. Taking advantage of these deletion events, we can recycle the selectable marker to create double or triple mutants and reporter gene strains using the same vectors or others using the same selectable marker.

Beyond the development of a new, targeted gene disruption tool, the creation of OA-minus mutants via CRISPR-Cas9-mediated *Ssoah1* disruption in three distinct isolates allowed us to characterize the loss of *Ssoah1* function phenotype in different genetic backgrounds. *Ssoah1* gene deletion and gene disruption mutants for two of the isolates (“1980” and “WMA,” respectively) were previously characterized (5, 6). Pathogenicity phenotypes for both sets of these previously reported mutants were remarkably similar in that they caused small limited lesions on numerous hosts, including *Arabidopsis*, soybean, common bean, and tomato. The previous characterization of the “WMA” *Ssoah1* mutants showed that inoculation of the cool-season legume hosts, faba bean and sugar snap peas, did not produce limited lesions but rather spreading lesions (6). Our results confirmed these infection phenotypes in the original “WMA” *Ssoah1* gene deletion mutant as well as in the CRISPR-Cas9-mediated disruption mutants derived from “WMA,” “1980,” and “UF1.” The original “1980” *Ssoah1* gene deletion mutant had pleiotropic phenotypes, including the absence of compound appressorium development not related directly to the loss of *Ssoah1* function (5). This conclusion is confirmed here in that none of 38 CRISPR-Cas9 *Ssoah1* mutants, including those derived from “1980,” were defective in compound appressorium production. To the contrary, all CRISPR-Cas9-mediated *Ssoah1* mutants displayed enhanced compound appressorium development. The enhanced production of compound appressorium development reported here is also a characteristic of the original “WMA” *Ssoah1* gene deletion mutant and thus not associated with the specific process used to generate the mutants. In all, three phenotypes associated with loss of *Ssoah1* function that had not been previously reported were observed in this study: (i) the overproduction of compound appressoria, (ii) decreased pigmentation on the surface of sclerotia, and (iii) a diffuse pattern of sclerotium development in culture. These findings demonstrate that the production of multiple mutants across multiple isolates can reveal phenotypes conserved within the species.

In addition to the *Ssoah1* locus, this CRISPR-Cas9 system was also used to disrupt *Sspks13*. Characterization of the resulting mutants confirmed the locus-independent nature of inserting rearranged vector sequences at DSBs using a single CRISPR-Cas9-Hyg plasmid in S. sclerotiorum. In Botrytis cinerea, the orthologous *bcpks13* and a second *bcpks12* (orthologous to *Sspks12*) encode the key polyketide synthases responsible for melanin biosynthesis (40, 41). The *bcpks13* gene is required for 1,8-dihydroxynaphthalene melanin accumulation in conidia, and *bcpks12* is required for melanin accumulation in sclerotia of B. cinerea (40). As S. sclerotiorum does not produce conidia but does produce compound appressoria which accumulate an amorphous, dark matrix surrounding them, we hypothesized that *Sspks13* may encode the polyketide synthase responsible for its accumulation. The hypothesis that *Sspks13* was responsible for melanin accumulation in compound appressoria was supported by the lack of compound appressorium pigment accumulation in the *Sspks13* disruption mutants created in both the S. sclerotiorum “1980” and “WMA” backgrounds at two independent target sites. The role of the orthologous *bcpks13* in B. cinerea compound appressorium pigmentation has not been reported, but similarly to the *Sspks13* mutants reported here, loss-of-function *bcpks13* mutants are fully infectious (40). The lack of a penetration phenotype for the S. sclerotiorum albino compound appressoria is consistent with the observations that melanin accumulation is not localized to a discrete wall layer of compound appressoria and thus not expected to play a role in turgor generation as in P. oryzae (42). However, given the structural and antioxidant roles of melanin in numerous biological systems, an infection-related phenotype might be expected. Perhaps, when examined under more natural infection conditions with variable environmental parameters or across an even greater variety of hosts, a function may be realized.

In conclusion, S. sclerotiorum is amenable to functional gene characterization via CRISPR-Cas9 technology. A key characteristic of the system reported here is that introduction of a single circular plasmid can produce high-efficiency insertion of linearized, rearranged vector sequences at the CRISPR-Cas9 target site likely mediated by an endogenous nuclease(s) and the NHEJ repair pathway. Mutants created with these described procedures are specific with no detected off-target sequence insertions or pleiotropic phenotypes. The mutant phenotypes are vegetatively stable in several independent mutants created in three wild-type backgrounds. The integration of multiple rearranged vector sequences at individual target sites facilitates the recovery of progeny lacking the selectable transformation marker via meiosis-associated intrachromosomal recombination. This increased capacity for rapid, single-molecule gene disruption vector construction and specific targeted gene disruption has the potential to advance the state of functional genomics in this important plant-pathogenic fungus.

## MATERIALS AND METHODS

### Fungal strains, culture conditions, and plant materials.

Mutants were derived from three wild-type S. sclerotiorum isolates: “1980” (“UF70”), isolated from bean culls in western Nebraska and fully genome sequenced (2); “UF1,” isolated from diseased petunia in Florida; and “WMA,” isolated from diseased pea in Washington state (6).

Cultures were routinely grown on potato dextrose agar (PDA) (Becton, Dickinson and Company, Franklin Lakes, NJ) at room temperature (22 to 24°C). Transformation mutants were cultured on PDA or regeneration medium (RM; 239.6 g sucrose, 0.5 g yeast extract, 15 g agar/liter or 8 g/liter agar for top agar) supplemented with 100 µg/ml hygromycin B (HygB) (EMD Biosciences, USA) and 50 µg/ml bromophenol blue (Sigma-Aldrich, USA) where indicated. Hypha stocks were stored on mycelium-colonized and desiccated filter paper or as dry sclerotia at −20°C. Ascospores were collected from apothecia of mature sclerotia produced in cultures grown on sterile smashed potato medium (SPM) (200 g of mashed potatoes with 1.5% agar) at room temperature (22 to 25°C). Apothecia were induced from SPM culture-derived sclerotia using the method of Li and Rollins (43).

Plants were grown in the glasshouse under natural sunlight, with a temperature range of 16 to 30°C. Yellow onions were purchased from a local grocery store. Seeds from Glycine max (soybean DP 2330 RR), Brassica carinata (genotype 110996 EW), Solanum lycopersicum (tomato cv. Better Boy), Vicia faba (faba bean cv. Windsor), and Pisum sativum (pea cv. Sugar Daddy) (Fig. 6 and 7) were planted in SunGro Metro-Mix 830 (SunGro Horticulture, Agawam, MA) potting soil and grown in 10-cm plastic pots in the glasshouse. Arabidopsis thaliana (*Arabidopsis* ecotype Col-0) was grown in the lab in the same potting soil in 5-cm plastic pots, under fluorescent lighting (12-h light/12-h dark) at 22 to 25°C.

### Construction of CRISPR-Cas9 system and verification of targeted mutagenesis.

All PCR primer sequences used in this study are shown in Table S1 in the supplemental material. The CRISPR-Cas9-TrpC-Hyg vector (Fig. S1) was derived from plasmid pCRISPR-Cas-TrpC (22) by integrating the hygromycin phosphotransferase (*hph*) marker for hygromycin B resistance (HygR). The *hph* cassette containing the TrpC promoter and terminator was cloned from a pNDH-OGG template (44). Primers Hyg-P-F and Hyg-T-R were used to amplify the *hph* cassette by adding XbaI enzyme sites and then ligating it into the CRISPR-Cas-TrpC (22) vector to obtain the selectable vector pCRISPR-Cas9-TrpC-Hyg. The modified vector was then used for single guide RNA (sgRNA) construction as previously described (33).

The *Ssoah1* gene (5) was selected as the initial mutagenesis target (GenBank accession number XM_001590428). The sgRNA primers for target sites within the *Ssoah1* locus were designed using the online E-Crispr tool (45) and screened for unique occurrence within the S. sclerotiorum “1980” genome (2) using BLASTN. The one-step Golden Gate cloning method was used to insert the target oligonucleotides (protospacer), as previously described (22). All sgRNA constructs were confirmed by PCR using Fsg forward primers for each protospacer and sgRNA-R located in its TrpC promoter. A total of 5 to 10 µg of each construct was used to transform S. sclerotiorum protoplasts using HygB selection on RM as described previously (46). The *Sspks13* gene was selected as the second target with locus identification number XM_001585755. The sgRNA primers (Table S1) were used, and cloning, transformation, and screenings for *Sspks13* were performed as described above for *Ssoah1*.

Primary verification of *Ssoah1* mutants was done after three hypha tip transfers on PDA medium supplemented with 100 µg/ml HygB. Medium acidification by OA was indicated by a violet-to-yellow color change in PDA medium (pH 7.0) supplemented with 50 µg/ml bromophenol blue (BPB). PCR was performed to amplify the *Ssoah1* fragment for primer-based confirmation of the mutation using F and R primers for targets 3, 4, 5, 7, 8, and 9 or F6 and R6 primers for target 6 (Table S1). Genomic DNA used as a template was isolated with the DNA minipreparation protocol (47). *Taq* DNA polymerase (New England BioLabs [NEB], Ipswich, MA) and LongAmp *Taq* DNA polymerase (NEB, Ipswich, MA) were used in combination with standard PCR components and standard programs used for PCR identification of the *Ssoah1* disruptions. PCR was also used for *Sspks13* mutant characterization using primers pks13-F and pks13-R.

### Identification of insertion site sequences by TAIL-PCR.

CRISPR-Cas9-mediated insertion site sequences were identified by thermal asymmetrical interlaced PCR (TAIL-PCR). The stock concentration of the primers used in TAIL-PCR was 10 µM, and the programs used for the TAIL-PCR were described previously (48). For recovery of the 5′ and 3′ insertion sequences, three nested primer pairs located in the *Ssoah1* locus were used in combination with two arbitrary primers, LAD1-4 and AC-1 (Table S1). To perform a primary PCR for 5′ sequences of the insertions, 3 ng/µl of genomic DNA was used with 0.4 μmol/liter F-SP1 and 1.4 μmol/liter LAD1-4. The resulting product from the initial PCR (1 µl) was used in the secondary reaction with 0.4 μmol/liter F-SP2 and AC-1 primers. Identical quantities of primers were used in both the secondary and tertiary PCRs. The tertiary TAIL-PCR products were purified from an 0.8% agarose gel using the Qiaex II gel extraction kit (Qiagen, Germantown, MD; catalog no. 20021) and then submitted for sequencing. For 3′ sequence insertion characterization, the specific primers R-SP1, R-SP2, and R-SP3 were used for each reaction.

### HygB resistance assay of mutants and their ascospore progeny.

For HygB resistance tests, the wild type, the *Ssoah1* disruption strains, and the empty vector transformants were grown on PDA medium with 100 mg/liter HygB. A HygB resistance test of ascospore progeny was done, allowing ascospores to germinate on PDA medium without HygB. Ascospore progeny were subsequently screened on PDA with 50 mg/liter BPB and on PDA supplemented with 100 mg/liter HygB for OA-minus and hygromycin B resistance confirmation, respectively. Genomic DNA was extracted as described above. LongAmp *Taq* DNA polymerase (NEB) was used for PCR amplification across insertion sites in ascospore progeny. To characterize the *hph* cassette deletion, three different pairs of primers were used to test amplification of the entire *hph* cassette from promoter to terminator (Hyg-P-F and Hyg-T-R), the promoter and coding sequence (Hyg-P-F and Hyg-C-R), and only the *hph* coding sequence (Hyg-C-F and Hyg-C-R). Primers Pycf and PycR designed to amplify *Sspyc1* (GenBank accession no. XM_001586211) were used as a positive control to verify PCR amplification template quality.

### Sequencing and analysis of the UF1-*oah1*-3-6 genome.

Genomic DNA was isolated from the UF1-*oah1*-3-6 mutant using the OmniPrep kit for fungi (G-Biosciences, USA). Paired-end sequencing (2 by 250) was conducted on a MiSeq sequencer (Illumina, San Diego, CA) by the Interdisciplinary Center for Biotechnology Research (ICBR; University of Florida, Gainesville, FL). A *de novo* genome assembly from paired-end reads was generated using SPAdes 3.10 (49) with default settings. Contigs containing vector sequences were identified by BLASTN search. Paired-end reads were aligned to each contig containing vector sequences with Bowtie 2 (default options), sorted, and used for coverage calculation with the SAMtools “depth” option. Contig order was predicted by contig splicing in DNAMAN 6 software (Lynnon Biosoft, USA). Large insertion sequences in ascospores after meiosis were amplified by LongAmp *Taq* polymerase (NEB), and the UF1-*oah1*-3-6-② *Ssoah1* insertion was sequenced after amplification by PCR. Global alignment of transformation vector sequences, parental mutant UF1-*oah1*-3-6, and single ascospore progeny UF1-*oah1*-3-6-② insertion sequences was performed with the Artemis comparison tool (ACT) (50). A series of PCR amplifications were also conducted to verify the large insertions, based on the splicing prediction and ACT analysis.

### OA-minus mutant identification and development of sclerotia.

Yeast-phosphate-sucrose (YPSu) liquid medium, pH 7.0, was used for pH testing and OA quantification as described previously (5). Three mycelial agar plugs were cultured on YPSu medium, and pH was quantified using pH indicator strips (pH 0 to 6; EMD, Germany). A 100-µl aliquot of culture filtrates was sampled at 3 DAI. OA quantification was performed using an oxalate assay kit (Sigma-Aldrich, USA). The morphological characteristics of S. sclerotiorum on YPSu were observed and photographed daily.

The wild type, the *Ssoah1* disruption mutant, and the empty vector transformants were grown on PDA medium in 9-cm petri dishes. Development and distribution of sclerotia were recorded at 10 DAI. Sclerotia were photographed and collected at that time.

### Appressorium development and pathogenicity tests.

Production of compound appressoria was done using fresh mycelial plugs (5-mm diameter) with growing hyphal tips. Plugs were placed on paraffin film (Parafilm M; Bemis NA, Neenah, WI) and incubated in a humidity chamber for 3 days. Compound appressoria could be observed macroscopically by the presence of pigmented development surrounding the agar plug. To quantify pigmented compound appressoria, ImageJ 1.50i software (https://imagej.nih.gov/ij/) was used to conduct particle quantification based on image contrast. Compound appressoria for macro- and microscopic observations were also produced on GelAir cellophane (Bio-Rad, Hercules, CA). For this, four agar plugs from PDA cultures were overlaid with cellophane and incubated for 3 days. Samples of the cellophane were removed and observed by light microscopy. For pathogenicity analysis, freshly collected leaflets from several host plants were inoculated with agar plugs taken from the edge of 1- to 2-day-old cultures on PDA and incubated at room temperature. Three to five leaflets of each plant species were inoculated with each strain. The experiment was repeated three times. Timing and symptom development were recorded. Lesion area was measured by ImageJ as described above. All statistical analyses were performed using PASW Statistics 18 (IBM, Inc.), and multiple comparisons of the means were carried out by one-way analysis of variance (ANOVA) with *post hoc* contrasts by Dunnett’s (2-sided) test.
