# Supplementary figures and images for: Introduction of Large Sequence Inserts by CRISPR-Cas9 To Create Pathogenicity Mutants in the Multinucleate Filamentous Pathogen Sclerotinia sclerotiorum
Source: mBio. 2018 Jun 26;9(3):e00567-18. doi: 10.1128/mBio.00567-18 (PMC6020291; doi:10.1128/mBio.00567-18)

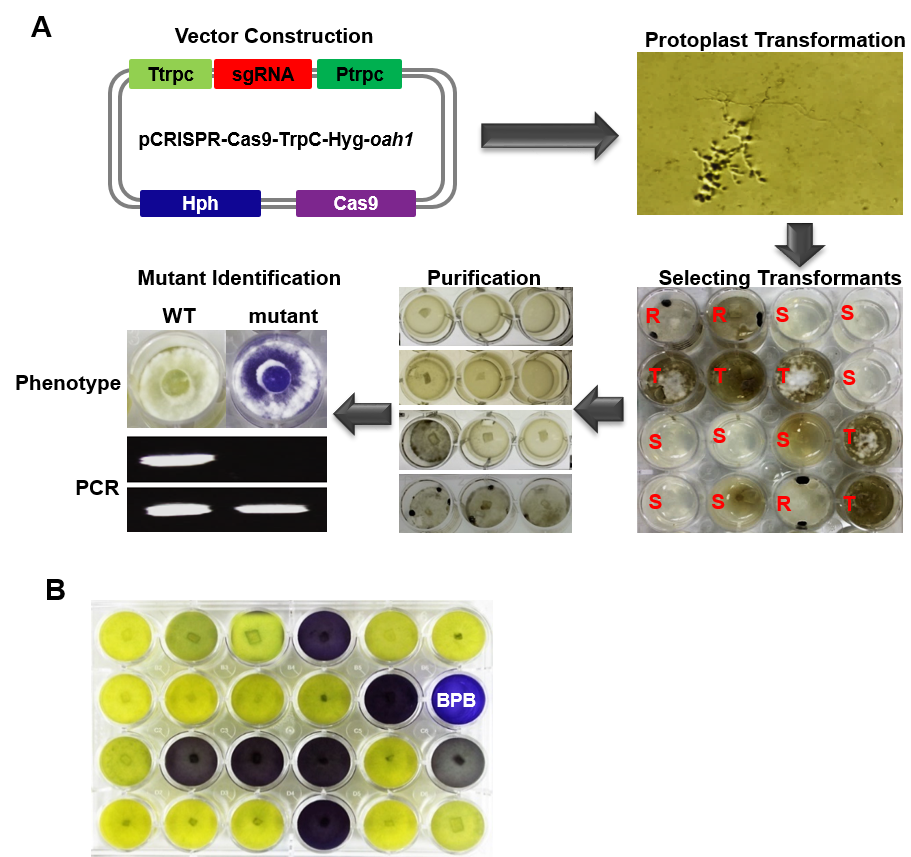

Supplement: FIG S1 [file mbo003183955sf1.tif]

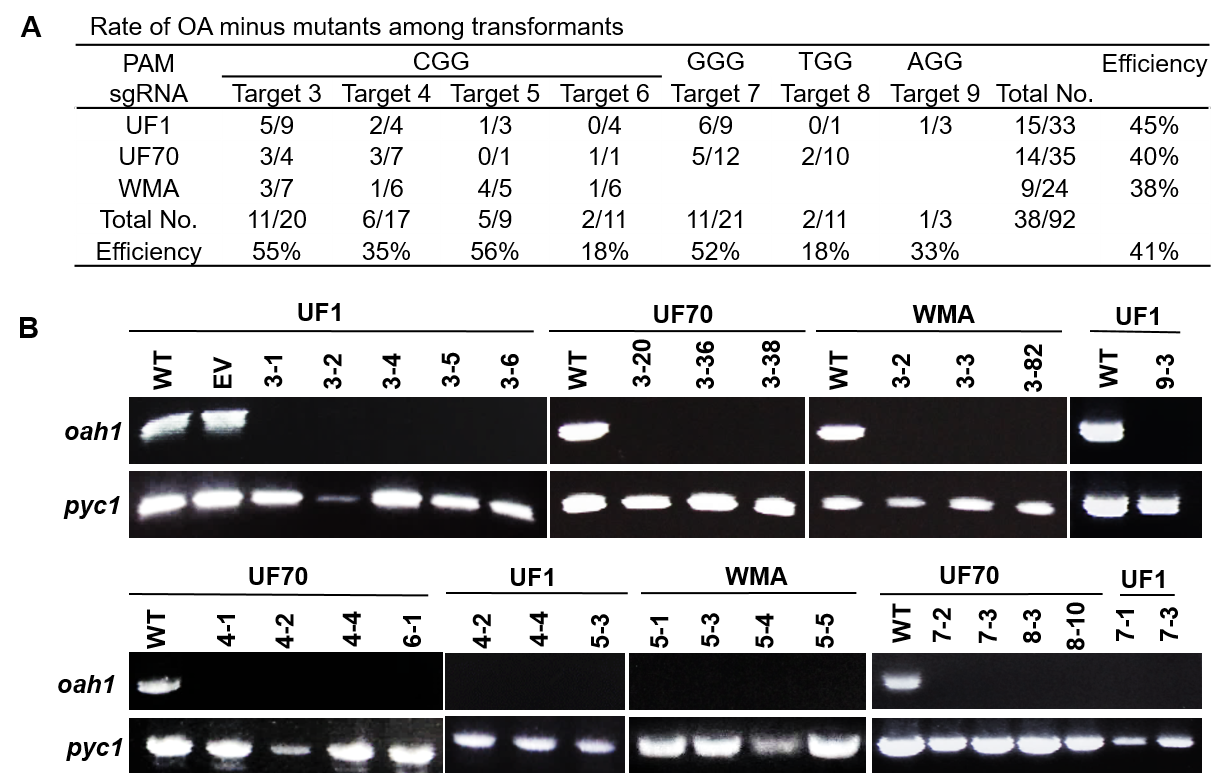

Supplement: FIG S2 [file mbo003183955sf2.tif]

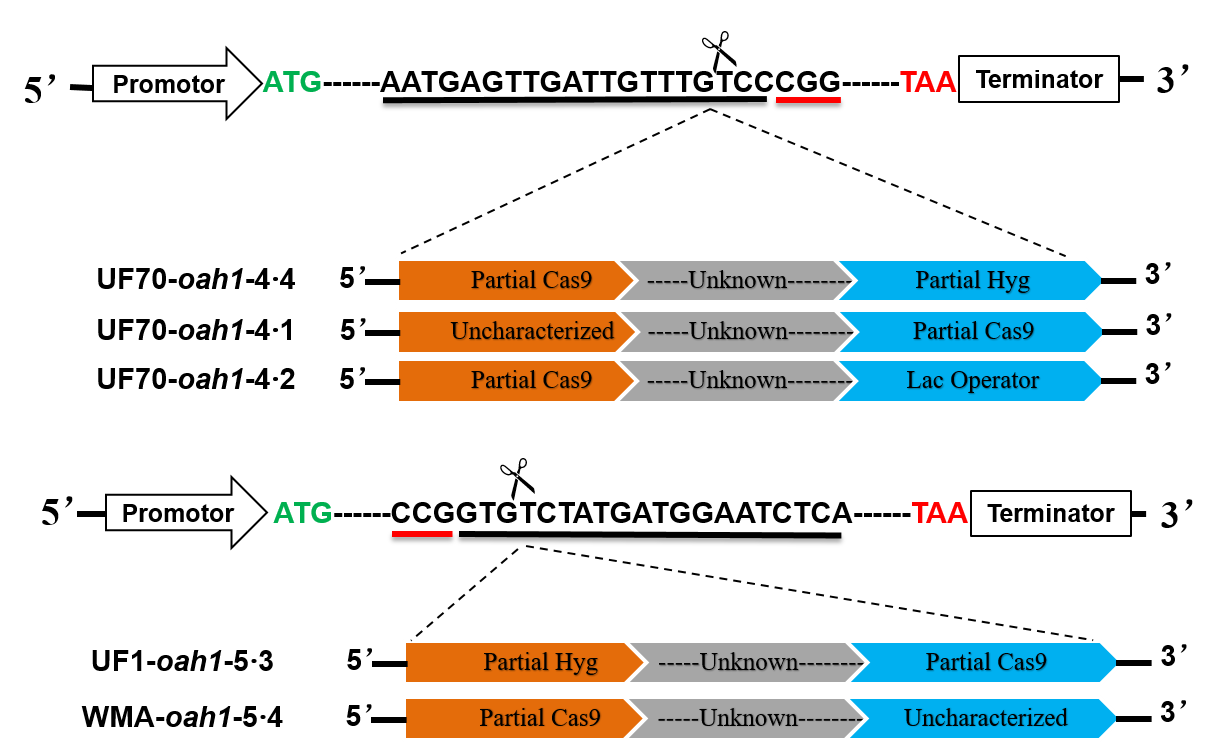

Supplement: FIG S3 [file mbo003183955sf3.tif]

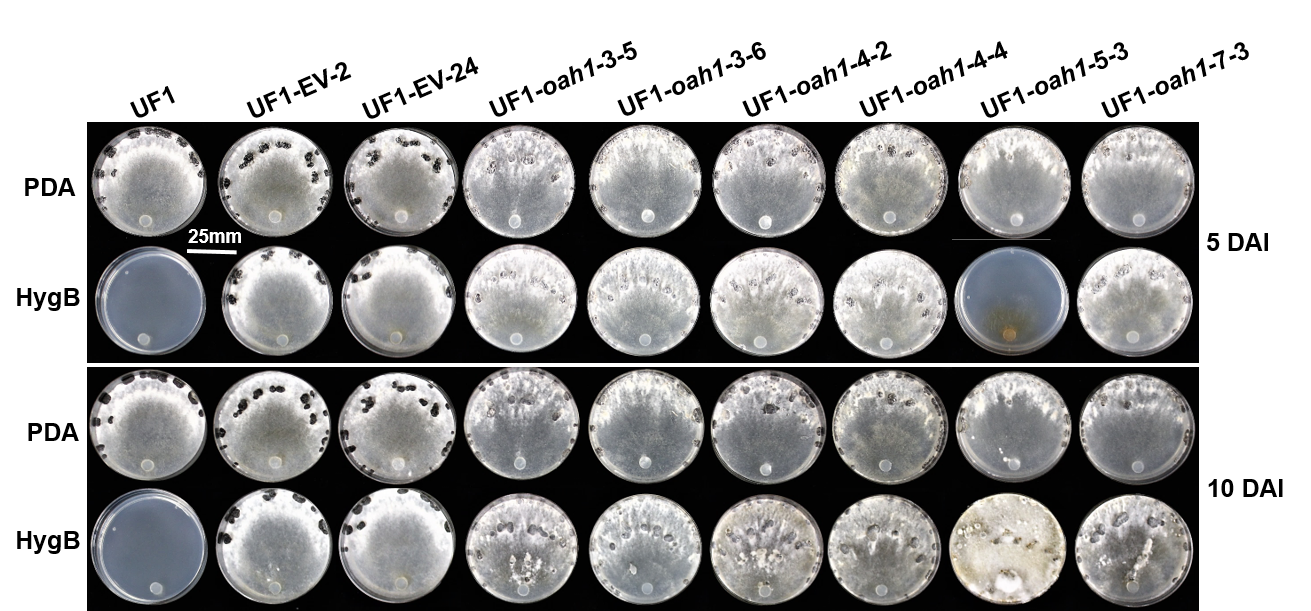

Supplement: FIG S4 [file mbo003183955sf4.tif]

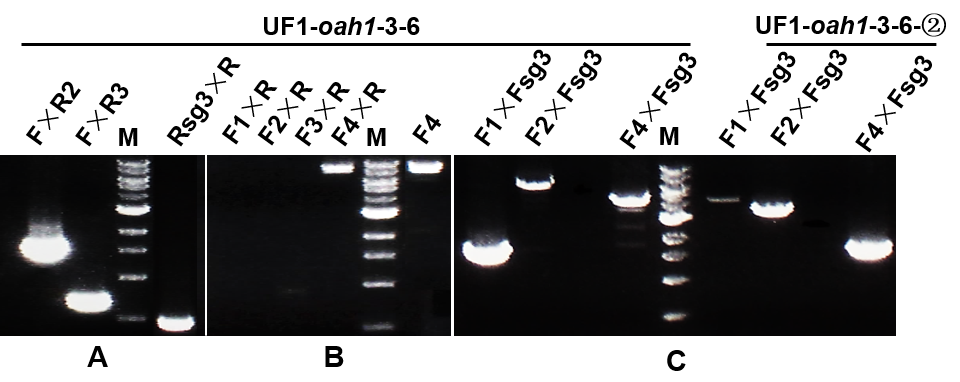

Supplement: FIG S5 [file mbo003183955sf5.tif]

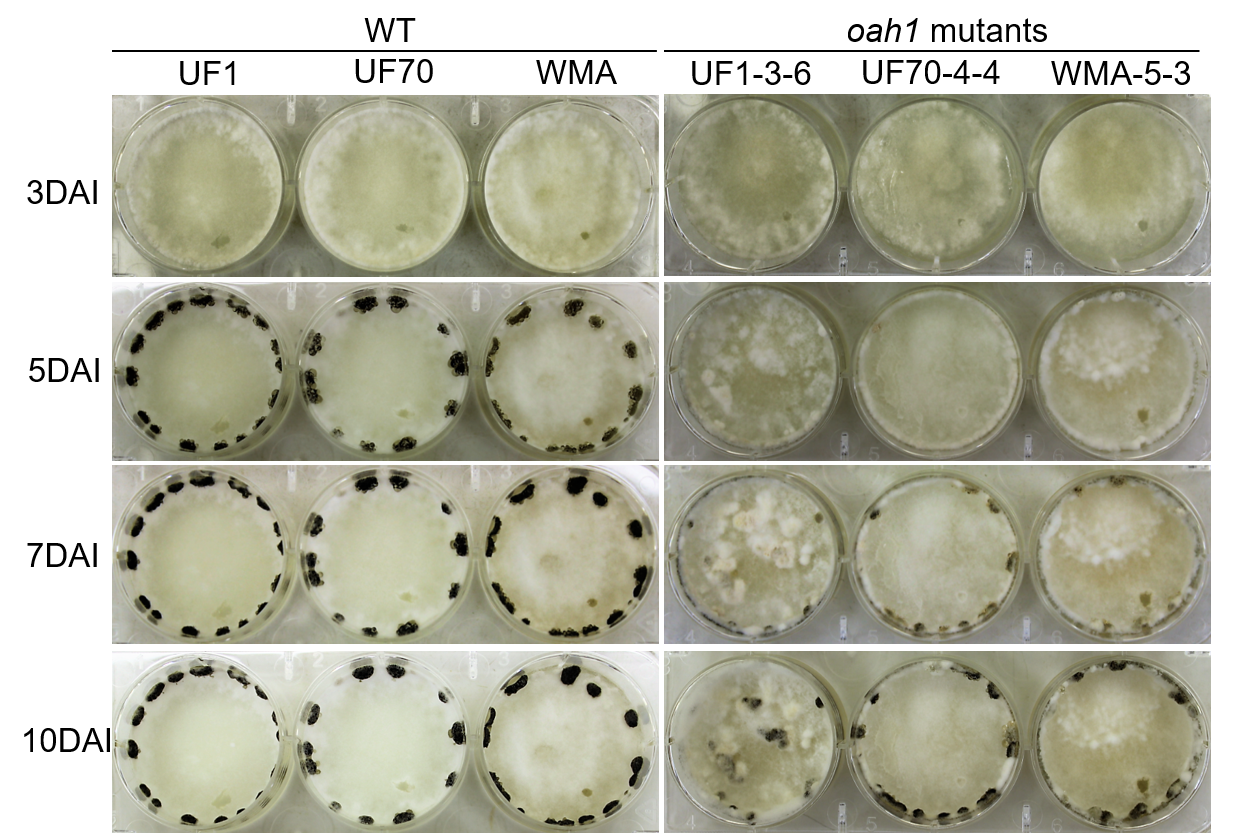

Supplement: FIG S6 [file mbo003183955sf6.tif]

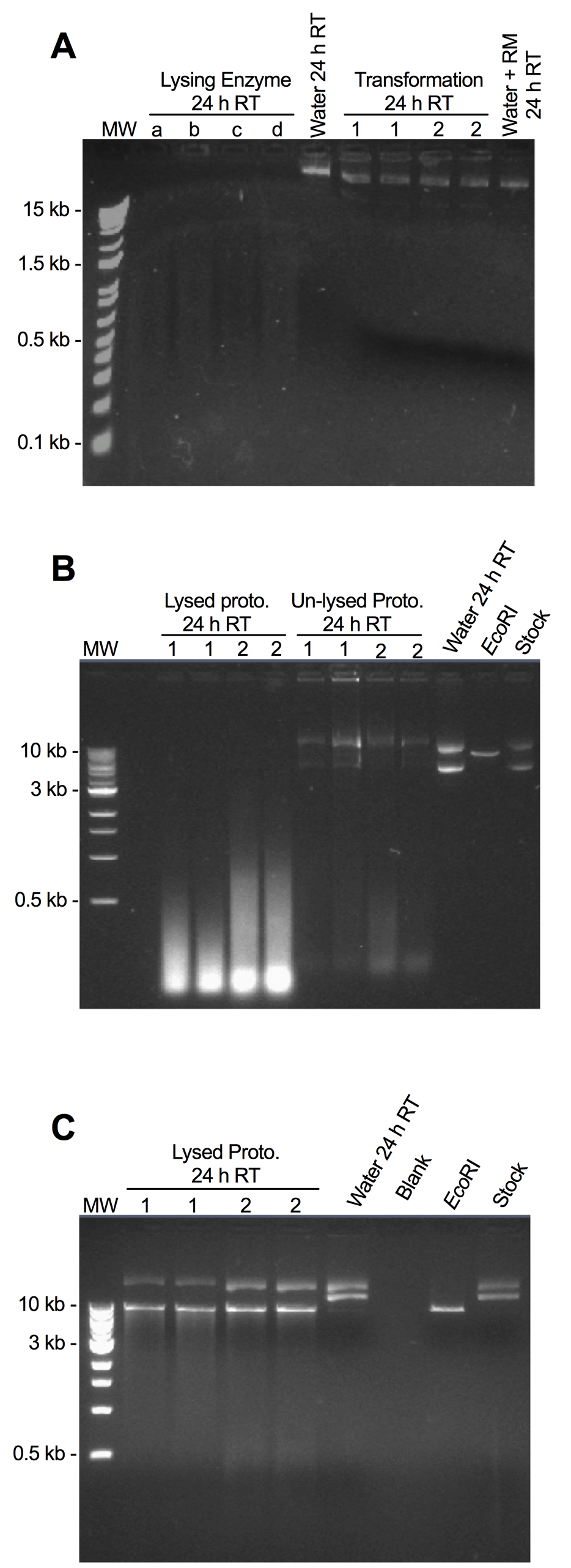

Supplement: FIG S7 [file mbo003183955sf7.tif]
