# Supplementary material for: Introduction of Large Sequence Inserts by CRISPR-Cas9 To Create Pathogenicity Mutants in the Multinucleate Filamentous Pathogen Sclerotinia sclerotiorum
Source: mBio. 2018 Jun 26;9(3):e00567-18. doi: 10.1128/mBio.00567-18 (PMC6020291; doi:10.1128/mBio.00567-18)
Supplement: TABLE S1 [file mbo003183955st1.docx]

Table S1 Primers used in this research

| Primer name | Sequence | Description |
| --- | --- | --- |
| Fsg3 | 5’- aaacGTGCCACCAAGTTGAAGAAC-3’ | Forward primer for target 3 sgRNA |
| Rsg3 | 5’-agttGTTCTTCAACTTGGTGGCAC-3’ | Reverse primer for target 3 sgRNA |
| Fsg4 | 5’-aaacGGACAAACAATCAACTCATT-3’ | Forward primer for target 4 sgRNA |
| Rsg4 | 5’-agttAATGAGTTGATTGTTTGTCC-3’ | Reverse primer for target 4 sgRNA |
| Fsg5 | 5’-aaacGTGTCTATGATGGAATCTCA-3’ | Forward primer for target 5 sgRNA |
| Rsg5 | 5’-agttTGAGATTCCATCATAGACAC-3’ | Reverse primer for target 5 sgRNA |
| Fsg6 | 5’-aaacCCAAGGGTGCCAAAGATGCC-3’ | Forward primer for target 6 sgRNA |
| Rsg6 | 5’-agttGGCATCTTTGGCACCCTTGG-3’ | Reverse primer for target 6 sgRNA |
| Fsg7 | 5’-aaacGGTGTCTATGATGGAATCTC-3’ | Forward primer for target 7 sgRNA |
| Rsg7 | 5’-agttGAGATTCCATCATAGACACC-3’ | Reverse primer for target 7sgRNA |
| Fsg8 | 5’-aaacGCTCTTTACATGGTACGTTC-3’ | Forward primer for target 8 sgRNA |
| Rsg8 | 5’-agttGAACGTACCATGTAAAGAGC-3’ | Reverse primer for target 8 sgRNA |
| Fsg9 | 5’-aaacCGACGATTGGGAGTGCATCC-3’ | Forward primer for target 9 sgRNA |
| Rsg9 | 5’-agttGGATGCACTCCCAATCGTCG-3’ | Reverse primer for target 9 sgRNA |
| sgRNA-R | 5’-ATCTGAACCATCCTTTGACCACCGTTTGCT-3’ | Reverse primer for sgRNA construction verification |
| F | 5’-GATACAGTAGATTGATGCGGG-3’ | Forward primer for target 3,4,5,7,8,9 mutant verification |
| R | 5’-GTCGGAAAGATGAGCAATG-3’ | Reverse primer for target 3,4,5,7,8,9 mutant verification |
| F6 | 5’-GAAGGTTGTCCCTGAAGAAGAG-3’ | Forward primer for target 6 mutant verification |
| R6 | 5’-CCTCCTTGGTGGTGATGATAG-3’ | Reverse primer for target 6 mutant verification |
| PycF | GCA AGG ATT CTC ACC AGA TAC | Forward primer for *pyc1* gene as control |
| PycR | CCC AAC ATT CCA AAC TGA AC | Reverse primer for *pyc1* gene as control |
| LAD1-4 | 5’-ACGATGGACTCCAGAGBDNBNNNGGTT-3’ | Arbitrary degenerate (AD) primers for the 1st tail PCR |
| AC-1 | 5’-ACGATGGACTCCAGAG-3’ | AD primers for the 2nd and 3rd tail PCR |
| F-SP1 | 5’-CTCACTCTGCGAGATGATACAT-3’ | Specific primer for the 5’ flank in the 1^st^ tail PCR |
| F-SP2 | 5’-GATACAGTAGATTGATGCGGG-3’ | Specific primer for the 5’ flank in the 2^nd^ tail PCR |
| F-SP3 | 5’-ATGGCTCCCATCATGGAT-3’ | Specific primer for the 5’ flank in the 3^rd^ tail PCR |
| R-SP1 | 5’-GTGCGTGCAATCAACACAATA-3’ | Specific primer for the 3’ flank in the 1^st^ tail PCR |
| R-SP2 | 5’-GTCGGAAAGATGAGCAATG-3’ | Specific primer for the 3’ flank in the 2^nd^ tail PCR |
| R-SP3 | 5’-CCAACTTGAAGGGCAACT-3’ | Specific primer for the 3’ flank in the 3^rd^ tail PCR |
| Hyg-P-F | GCTCTAGAGATATTGAAGGAGCATTTTTTGGGC | Forward primer for hph promotor (*Xba*I, underline) |
| Hyg-T-R | GCTCTAGACGCATTGGATTAATAATTGTTGCTA | Reverse primer for hph terminator (*Xba*I, underline) |
| Hyg-C-F | GCTCTCGGAGGGCGAAGAAT | Forward primer for hph coding sequence |
| Hyg-C-R | CTATTCCTTTGCCCTCGGACGAGTGCT | Reverse primer for hph coding sequence |
| F1 | GAACTACTGGCGTCAGTTGC | Forward primer for 3-6 and 3-6-2 insertion |
| F2 | GGCTAAGGGCTATAAGGAAG | Forward primer for 3-6 and 3-6-2 insertion |
| F3 | TGTGTGAAATTGTTATCCGC | Forward primer for 3-6 and 3-6-2 insertion |
| F4 | GTCTGACGCTCAGTGGAACG | Forward primer for 3-6 and 3-6-2 insertion |
| F5 | TGCGCCCACTCACTCACACTC | Reverse primer for 3-6 and 3-6-2 insertion |
| R2 | GAGCAACTGACGCCAGTAGTTCT | Reverse primer for 3-6 and 3-6-2 insertion |
| R3 | GACTTCCTTATAGCCCTTAGCCTC | Reverse primer for 3-6 and 3-6-2 insertion |
| pks13-Fsg4 | 5’-aaacGGTCCAAAAGCTCGAACACC-3’ | Forward primer for target 4 sgRNA of Sspks13 |
| pks13-Rsg4 | 5’-agttGGTGTTCGAGCTTTTGGACC-3’ | Reverse primer for target 4 sgRNA of Sspks13 |
| pks13-Fsg5 | 5’-aaacTATTCGCAGGCGGTATGAAT-3’ | Forward primer for target 5 sgRNA of Sspks13 |
| pks13-Rsg5 | 5’-agttATTCATACCGCCTGCGAATA-3’ | Reverse primer for target 5 sgRNA of Sspks13 |
| pks13-F | 5’-GAAATCCCTGCCGATAGAT-3’ | Forward primer for target 4 and 5 mutant verification |
| pks13-R | 5’-TGGTGTTGGTTTGAAGGC-3’ | Reverse primer for target 4 and 5 mutant verification |
