# Supplementary material for: Introduction of Large Sequence Inserts by CRISPR-Cas9 To Create Pathogenicity Mutants in the Multinucleate Filamentous Pathogen Sclerotinia sclerotiorum
Source: mBio. 2018 Jun 26;9(3):e00567-18. doi: 10.1128/mBio.00567-18 (PMC6020291; doi:10.1128/mBio.00567-18)
Supplement: TEXT S1 [file mbo003183955s1.pdf]

Supplemental Figure S7 Materials and Methods

Restriction enzyme digestion

Plasmid DNA (pCRISPR-Cas-TrpC-Hyg-oah1-3) was digested with restriction enzyme EcoRI (New England Biolabs, MA, USA) which recognizes a single restriction site in the plasmid sequence. The 100 µl reaction mixture contained 400 ng of plasmid DNA, 10 µl of 10X NEBuffer, and 1 µl of EcoRI (20 units/µl). The mixture was incubated at 37°C for one hour.

Lysing enzyme mixture preparation

To prepare the lysing enzyme mixture, 200 g of lysing enzyme (Sigma-Aldrich, MO, USA) was added into 3 ml of Novozyme buffer (1 M Sorbitol, 50 mM Sodium citrate, pH 5.8) and filter sterilize with 0.45 µm filter, followed by the addition of 17 ml of protoplast buffer (0.8 M $MgS0_4 \cdot 7H_2O$, 0.2 M Sodium citrate• $2H_2O$, pH 5.5).

Detection of nuclease activity

Protoplast prepared from *Sclerotinia sclerotiorum* isolates UF-1 and 1980 were used to assay DNA degraded by exogenous or endogenous nuclease during the transformation process. pCRISPR-Cas9-TrpC-oah1-3 plasmid was extracted with Wizard® Plus SV Minipreps DNA Purification Systems (Promega, WI, USA). To test the exogenous nuclease activity in the lysing enzyme mixture, 6 µg of plasmid DNA was incubated with 1 ml of lysing enzyme for 24hr at room temperature. The control was plasmid incubated in nuclease free water at the same concentration and conditions. To test for exogenous nucleases introduced during the genetic transformation process, plasmid DNA was carried through the PEG-mediated transformation protocol and sampled at various points along the procedure. As no substantial degradation was observed, only the sample from the last step of transformation (following 24 h incubation in Regeneration Medium) is presented. In detail: 6 µg of plasmid DNA was incubated with 4 µl spermidine (50mM) and 10 µl heparin (5 mg/ml in STC) and kept on ice for 20 min, then 100 µl of protoplasts ($1x10^8$/ml) was added into the DNA suspension and kept on ice for 30 min. Next, 1 ml of 40% polyethylene glycol (PEG) solution [60% w/v PEG:KTC buffer (1.8 M KCl, 150 mM Tris, pH 8, 150 mM $CaCl_2$) = 3:1] was added into the DNA-protoplast mixture and kept at room temperature for 30 min before addition of 1 ml of liquid regeneration medium (RM) (0.7 M sucrose, and 0.5 g yeast extract). This mixture was kept at room temperature with slow shaking (100 rpm) for 24 h. To test for endogenous nuclease activity, 6 µg of plasmid DNA was incubated with lysed and un-lysed protoplasts for 24 h at room temperature. Lysed protoplasts were prepared by pelleting 100µl of protoplast ($1x10^7$) at 3000 rpms for 5 min, removing the supernatant, and re-suspending in nuclease-free water. Un-lysed protoplast followed the same procedure but were resuspended in the original storage buffer. Uncut plasmid DNA incubated in nuclease free water under the same conditions and duration was used as a control. Following treatments, samples of each treatment and control were examined by agarose gel electrophoresis to assess the integrity of plasmid DNA.
